# Supplementary material for: Scaling-up a pharmacist-led information technology intervention (PINCER) to reduce hazardous prescribing in general practices: Multiple interrupted time series study
Source: PLoS Med. 2022 Nov 16;19(11):e1004133. doi: 10.1371/journal.pmed.1004133 (PMC9718399; doi:10.1371/journal.pmed.1004133)
Supplement: S1 Appendix — (PDF) [file pmed.1004133.s001.pdf]

S1 Appendix. Rollout of the PINCER intervention across general practices in 11 CCGs

		Q1 2015			Q2 2015/16			Q3 2016			Q4 2016			Q1 2016			Q2 2016/17			Q3 2017			Total (CCG)
CCG	Date first practice initiated	Aug	Sep	Oct	Nov	Dec	Jan	Feb	Mar	Apr	May	Jun	Jul	Aug	Sep	Oct	Nov	Dec	Jan	Feb	Mar	Apr	
1	25/09/2015		2	18	2					1													23
2	01/10/2015			35	13	1			1			2	2										54
3	16/10/2015			6	5	1																	12
4	30/10/2016			1	2	6	3																12
5	26/01/2016						2	3	3	3	3	3	1		1								19
6	16/02/2016							4	25	4	2												35
7	12/02/2016							2	13														15
8	14/04/2016									26	10	4	1		1	1							43
9	16/05/2016										4	9	5	18	9	6	6				1		58
10	01/07/2016												18	3	8				2				31
11	10/08/2016													1	13	7	17	2			1		41
Total (month)			2	60	22	8	5	9	42	34	19	18	27	22	32	14	23	2	2		2		
Total (quarter)		62			35			85			64			68			27			2			343

The cells contain the number of practices in each quarter that ran the first computer queries to identify patients at risk, as part of the PINCER intervention.
Q, quarter; CCG, Clinical commissioning group.
