# Supplementary material for: Scaling-up a pharmacist-led information technology intervention (PINCER) to reduce hazardous prescribing in general practices: Multiple interrupted time series study
Source: PLoS Med. 2022 Nov 16;19(11):e1004133. doi: 10.1371/journal.pmed.1004133 (PMC9718399; doi:10.1371/journal.pmed.1004133)
Supplement: S2 Appendix — (PDF) [file pmed.1004133.s002.pdf]

S2 Appendix. The Explicit statistical Model

Formal comparison utilised an interrupted time series approach, with binomial mixed models for the quarterly event numbers (*events*) with the appropriate denominator (*risk*), modelled as a categorical variable of the number of quarters since the intervention (i*QpostCode – coded as 1-pre, 2-iQ=0, 3-iQ=1,… where iQ is the number of quarters since implementation started, iQ=0 being the implementation quarter, so iQpostCode=1 is the reference level (pre-implementation). iQ takes negative values for pre-implementation quarters*), with all pre-intervention assigned a single level. Calendar time (*Qcal*) will be included as a covariate, modelled as a linear function of the calendar quarter. Random effect terms (random intercept for practice (*GP*) will allow for within practice correlations. Estimation of confidence intervals will utilise the standard robust SE approach.

$$Logit(P_{ij}) = \beta_0 + \ \beta cal \cdot Qcal_i + \beta post_i \cdot Qpost_i \ + u_{\ j} GP_{\ ij}$$

Where P_{ij} is the probability of a harm event in calendar quarter *i* and GP *j*. *Qpost* is zero for all pre-implementation quarters and u_j is a random intercept for each GP.

In Stata the model was specified as:

```
melogit events Qcal ib1.iQpostCode || GP: , binomial(risk) startvalues(constantonly) vce(cluster GP) or
```

where we specify *startvalues* to improve convergence and specify the robust SE in the *vce* option. Post treatment quarter is coded as above and parameter estimates are referenced to the pre-implementation quarter (*ib1*). The *or* option simple requests that the estimates be displayed exponentiated – ie as odds ratios.

Specific hypotheses of improvements over temporal trends at 6m,12m and 24m, corresponding to the assessment times in the previous cluster randomised trial, will be tested by constructing appropriate contrasts comparing the 2nd ,4th and 8th quarters (i*Q*=2,4,8 where *Qimp*=0 is the quarter during which the implementation was introduced) with the pre-implementation level.
