# Supplementary material for: Scaling-up a pharmacist-led information technology intervention (PINCER) to reduce hazardous prescribing in general practices: Multiple interrupted time series study
Source: PLoS Med. 2022 Nov 16;19(11):e1004133. doi: 10.1371/journal.pmed.1004133 (PMC9718399; doi:10.1371/journal.pmed.1004133)
Supplement: S3 Appendix — (PDF) [file pmed.1004133.s003.pdf]

STROBE Statement—checklist of items that should be included in reports of observational studies

	Item No.	Recommendation	Section and paragraph number	Relevant text from manuscript
**Title and abstract**	1	(*a*) Indicate the study's design with a commonly used term in the title or the abstract	Title<br><br>Abstract, Methods and findings, paragraph 1	Multiple interrupted time series design
		(*b*) Provide in the abstract an informative and balanced summary of what was done and what was found	Abstract	
**Introduction**				
Background/rationale	2	Explain the scientific background and rationale for the investigation being reported	Introduction, paragraphs 1-4	
Objectives	3	State specific objectives, including any prespecified hypotheses	Introduction, paragraphs 5	Objective- to assess whether PINCER was effective at reducing rates of hazardous prescribing when rolled out at scale across the East Midlands in the real world setting.<br><br>Hypothesis -the intervention would result in clinically important, sustained reductions in hazardous prescribing.
**Methods**				
Study design	4	Present key elements of study design early in the paper	Methods- Study design and participants, paragraph 1<br><br>Methods- Statistical methods, paragraph 1	
Setting	5	Describe the setting, locations, and relevant dates, including periods of recruitment, exposure, follow-up, and data collection	Setting/ location- Methods- Study design and participants, paragraph 2	

			Exposure- Methods- The Intervention, bullet point 1<br><br>Follow up/ data collection- Methods- Outcome measures, paragraph 1	
Participants	6	(*a*) *Cohort study*—Give the eligibility criteria, and the sources and methods of selection of participants. Describe methods of follow-up<br>*Case-control study*—Give the eligibility criteria, and the sources and methods of case ascertainment and control selection. Give the rationale for the choice of cases and controls<br>*Cross-sectional study*—Give the eligibility criteria, and the sources and methods of selection of participants	Methods- Study design and participants, paragraph 2	
		(*b*) *Cohort study*—For matched studies, give matching criteria and number of exposed and unexposed<br>*Case-control study*—For matched studies, give matching criteria and the number of controls per case	n/a	
Variables	7	Clearly define all outcomes, exposures, predictors, potential confounders, and effect modifiers. Give diagnostic criteria, if applicable	Outcomes- Methods- outcome measures, paragraph 2<br><br>Exposures- Methods- outcome measures, paragraph 1<br><br>potential confounders- Methods- Statistical Methods, paragraph 5	
Data sources/ measurement	8*	For each variable of interest, give sources of data and details of methods of assessment (measurement). Describe comparability of assessment methods if there is more than one group	Methods- The Intervention, paragraph 4	Methods- The Intervention, paragraph 4
Bias	9	Describe any efforts to address potential sources of bias	Results-Table 2	Characteristics of the practices included were compared to all

				English practices to check for bias
Study size	10	Explain how the study size was arrived at	Methods- Study design and participants. paragraph 2	All practices in the East Midlands were invited to participate.
Quantitative variables	11	Explain how quantitative variables were handled in the analyses. If applicable, describe which groupings were chosen and why	Methods- Outcome measures, paragraph 1 and 2	
Statistical methods	12	(*a*) Describe all statistical methods, including those used to control for confounding	Methods- Statistical methods, paragraphs 1 to 6	
		(*b*) Describe any methods used to examine subgroups and interactions	Methods- Statistical methods, paragraph 5	
		(*c*) Explain how missing data were addressed	Methods- Statistical methods, paragraph 3	
		(*d*) *Cohort study*—If applicable, explain how loss to follow-up was addressed<br>*Case-control study*—If applicable, explain how matching of cases and controls was addressed<br>*Cross-sectional study*—If applicable, describe analytical methods taking account of sampling strategy	Methods- Statistical methods, paragraph 2	
		(*e*) Describe any sensitivity analyses	Results, paragraph 1	As a sensitivity analysis, we also repeated the analysis including only practices with at least 6 months of follow up data.<br><br>We compared the raw and adjusted pre implementation rates for those practices with up to 6 months and up to 12 months follow up.
**Results**				
Participants	13*	(a) Report numbers of individuals at each stage of study—eg numbers potentially eligible, examined for eligibility, confirmed eligible, included in the study, completing follow-up, and analysed	Results, paragraph 1	PINCER was implemented in 370 practices and data were collected for 343 practices
		(b) Give reasons for non-participation at each stage	Results, paragraph 1	Ten practices had closed by the end of the implementation period, and 11 practices from one

				CCG were involved in piloting PINCER. A practice must have run the intervention and uploaded at least one quarter of data.
		(c) Consider use of a flow diagram	n/a	
Descriptive data	14*	(a) Give characteristics of study participants (eg demographic, clinical, social) and information on exposures and potential confounders	Results, Table 2	
		(b) Indicate number of participants with missing data for each variable of interest	Results, paragraph 1	At 6 months follow up, data for 212 practices were collected and by 12 months follow up, this had reduced to 70 (S4 Appendix).
		(c) *Cohort study*—Summarise follow-up time (eg, average and total amount)	n/a	
Outcome data	15*	*Cohort study*—Report numbers of outcome events or summary measures over time	S1 Appendix	
		Case-control study—Report numbers in each exposure category, or summary measures of exposure	n/a	
		Cross-sectional study—Report numbers of outcome events or summary measures	n/a	
Main results	16	(*a*) Give unadjusted estimates and, if applicable, confounder-adjusted estimates and their precision (eg, 95% confidence interval). Make clear which confounders were adjusted for and why they were included	Results, table 4	adjusted for general practice and calendar time
		(*b*) Report category boundaries when continuous variables were categorized	n/a	
		(*c*) If relevant, consider translating estimates of relative risk into absolute risk for a meaningful time period	n/a	
Other analyses	17	Report other analyses done—eg analyses of subgroups and interactions, and sensitivity analyses	Results, paragraph 1	As a sensitivity analysis, we also repeated the analysis including only practices with at least 6 months of follow up data.<br><br>We compared the raw and adjusted pre implementation rates for those practices with up to 6

				months and up to 12 months follow up.
**Discussion**				
Key results	18	Summarise key results with reference to study objectives	Discussion, paragraph 1-3	
Limitations	19	Discuss limitations of the study, taking into account sources of potential bias or imprecision. Discuss both direction and magnitude of any potential bias	Discussion, paragraph 9-12	
Interpretation	20	Give a cautious overall interpretation of results considering objectives, limitations, multiplicity of analyses, results from similar studies, and other relevant evidence	Discussion, paragraph 16	
Generalisability	21	Discuss the generalisability (external validity) of the study results	Discussion, paragraph 8	
**Other information**				
Funding	22	Give the source of funding and the role of the funders for the present study and, if applicable, for the original study on which the present article is based	Funding section	Health Foundation, London England; East Midlands

*Give information separately for cases and controls in case-control studies and, if applicable, for exposed and unexposed groups in cohort and cross-sectional studies.

Note: An Explanation and Elaboration article discusses each checklist item and gives methodological background and published examples of transparent reporting. The STROBE checklist is best used in conjunction with this article (freely available on the Web sites of PLoS Medicine at http://www.plosmedicine.org/, Annals of Internal Medicine at http://www.annals.org/, and Epidemiology at http://www.epidem.com/). Information on the STROBE Initiative is available at www.strobe-statement.org.
