# Supplementary material for: Scaling-up a pharmacist-led information technology intervention (PINCER) to reduce hazardous prescribing in general practices: Multiple interrupted time series study
Source: PLoS Med. 2022 Nov 16;19(11):e1004133. doi: 10.1371/journal.pmed.1004133 (PMC9718399; doi:10.1371/journal.pmed.1004133)
Supplement: S4 Appendix — (PDF) [file pmed.1004133.s004.pdf]

S4 Appendix. The number of practices included at each quarter, by time since the intervention

Quarter number	Freq.
-14	2
-13	6
-12	75
-11	142
-10	236
-9	258
-8	342
-7	343
-6	343
-5	341
-4	343

Quarter number	Freq.
-3	343
-2	342
-1	341
0	334
1	310
2	212
3	127
4	70
5	42
6	30
7	27
