# Supplementary material for: Scaling-up a pharmacist-led information technology intervention (PINCER) to reduce hazardous prescribing in general practices: Multiple interrupted time series study
Source: PLoS Med. 2022 Nov 16;19(11):e1004133. doi: 10.1371/journal.pmed.1004133 (PMC9718399; doi:10.1371/journal.pmed.1004133)
Supplement: S5 Appendix — (PDF) [file pmed.1004133.s005.pdf]

S5 Appendix. Comparing the rate of hazardous prescribing at 6 months and 12 months post-intervention to pre intervention, adjusted for GP practice and calendar time (excluding practices with less than 6 months of data post intervention)

	Raw data [a]		Pre-implementation	6 months		12 months	
Outcome	Number at risk	Rate [b]	Fitted rate (95% CI) [b, c]	Fitted rate (95% CI) [b, c]	OR (95% CI) [d]	Fitted rate (95% CI) [b, c]	OR (95% CI) [d]
Overall composite	581296	26.7	26.3 (26.2:26.3)	21.9 (21.8:22.0)	0.83 (0.80:0.86)	22.3 (22.2:22.3)	0.84 (0.80:0.89)
GI composite	356119	31.6	31.5 (31.5:31.6)	24.0 (24.0:24.1)	0.76 (0.72:0.79)	24.0 (23.9:24.1)	0.76 (0.70:0.82)
Indicator A	231450	27.4	25.6 (25.5:25.7)	18.7 (18.6:18.8)	0.72 (0.68:0.77)	17.4 (17.3:17.5)	0.67 (0.60:0.76)
Indicator B	15043	25.8	22.8 (22.7:22.9)	17.9 (17.7:18.1)	0.78 (0.67:0.91)	15.9 (15.6:16.1)	0.69 (0.55:0.87)
Indicator C	15043	88.2	81.4 (81.3:81.4)	65.0 (64.9:65.1)	0.78 (0.72:0.85)	60.8 (60.6:60.9)	0.73 (0.65:0.82)
Indicator D	31483	13.8	11.9 (11.8:12.0)	11.6 (11.4:11.7)	0.97 (0.86:1.10)	10.8 (10.7:11.0)	0.91 (0.78:1.06)
Indicator E	20155	40.7	38.9 (38.8:39.0)	27.4 (27.3:27.5)	0.70 (0.62:0.78)	30.8 (30.6:31.0)	0.78 (0.66:0.93)
Indicator F	42946	45.2	44.6 (44.5:44.6)	35.9 (35.8:35.9)	0.80 (0.74:0.86)	38.1 (38.0:38.2)	0.85 (0.75:0.97)
Indicator G	171795	9.1	7.8 (7.8:7.9)	7.7 (7.6:7.8)	0.98 (0.92:1.04)	8.2 (8.1:8.3)	1.04 (0.95:1.14)
Indicator H	3765	160.9	189.2 (189.1:189.4)	192.6 (192.4:192.7)	1.02 (0.91:1.15)	163.6 (163.4:163.9)	0.84 (0.67:1.05)
Indicator I	15220	22.3	19.8 (19.6:19.9)	17.4 (17.3:17.6)	0.88 (0.78:0.99)	16.1 (15.8:16.3)	0.81 (0.65:1.01)
Indicator J	13587	92.7	79.2 (79.1:79.3)	84.9 (84.8:85.0)	1.08 (1.00:1.17)	92.2 (92.1:92.3)	1.18 (1.05:1.33)
Indicator K	20811	24.5	20.9 (20.8:21.1)	19.1 (18.9:19.2)	0.91 (0.80:1.03)	16.8 (16.6:17.0)	0.80 (0.66:0.97)

GI, Gastrointestinal; OR, Odds ratio; CI, Confidence interval

[a] Number at risk and pre-intervention rates estimated as mean over the 4 quarters prior to intervention at each site

[b] Rate per 1000 patients at risk

[c] Fitted rates are adjusted for calendar time

[d] Odds ratio relative to pre-implementation
