# Supplementary material for: Scaling-up a pharmacist-led information technology intervention (PINCER) to reduce hazardous prescribing in general practices: Multiple interrupted time series study
Source: PLoS Med. 2022 Nov 16;19(11):e1004133. doi: 10.1371/journal.pmed.1004133 (PMC9718399; doi:10.1371/journal.pmed.1004133)
Supplement: S6 Appendix — (PDF) [file pmed.1004133.s006.pdf]

S6 Appendix. Comparing the baseline (pre-implementation year) rates of hazardous prescribing for practice with at least and without at least 6 months and 12 months post-intervention data: raw data and data adjusted for GP practice and calendar time

	All practices (n=343)		Practices with>=6 months of data post-implementation (n=212)		Practices with<6 months of data post-implementation (n=131)		Practices with>=12 months of data post-implementation (n=70)		Practices with<12 months of data post-implementation (n=273)	
Outcome	**Raw data Rate [a, b]**	**Fitted rate (95% CI) [b, c]**	**Raw data Rate [b]**	**Fitted rate (95% CI) [b, c]**	**Raw data Rate [b]**	**Fitted rate (95% CI) [b, c]**	**Raw data Rate [b]**	**Fitted rate (95% CI) [b, c]**	**Raw data Rate [b]**	**Fitted rate (95% CI) [b, c]**
**Overall composite**	26.4	27.2 (27.1:27.2)	26.7	27.3 (27.2:27.3)	25.6	27.0 (27.0:27.1)	25.9	26.2 (26.1:26.3)	26.5	27.4 (27.4:27.5)
**GI composite**	31.4	33.2 (33.1:33.2)	31.6	33.1 (33.0:33.1)	30.9	33.3 (33.2:33.3)	30.4	31.6 (31.5:31.7)	31.7	33.6 (33.5:33.6)
**Indicator A**	26.9	27.3 (27.2:27.3)	27.4	27.5 (27.4:27.6)	25.7	26.9 (26.8:27.0)	23.9	23.5 (23.3:23.7)	27.8	28.3 (28.3:28.4)
**Indicator B**	25.5	24.0 (24.0:24.1)	25.8	23.5 (23.4:23.6)	24.9	25.0 (24.9:25.2)	23.4	21.0 (20.8:21.2)	26.1	24.9 (24.8:25.0)
**Indicator C**	86.6	80.5 (80.5:80.6)	88.2	84.6 (84.6:84.7)	83.2	73.5 (73.4:73.6)	88.8	83.0 (82.8:83.1)	85.9	79.9 (79.8:80.0)
**Indicator D**	13.7	12.1 (12.0:12.1)	13.8	12.0 (11.9:12.1)	13.5	12.1 (12.0:12.3)	16.6	13.4 (13.2:13.6)	12.9	11.7 (11.6:11.8)
**Indicator E**	39.1	39.0 (38.9:39.0)	40.7	40.4 (40.4:40.5)	35.2	36.5 (36.4:36.6)	46.8	46.9 (46.8:47.1)	36.7	37.1 (37.1:37.2)
**Indicator F**	46.4	44.7 (44.7:44.7)	45.2	43.4 (43.3:43.4)	48.9	47.0 (46.9:47.1)	48.0	47.9 (47.8:48.0)	45.9	43.9 (43.9:44.0)
**Indicator G**	8.9	7.4 (7.3:7.4)	9.1	7.5 (7.4:7.6)	8.5	7.1 (7.0:7.3)	9.4	8.3 (8.2:8.4)	8.7	7.1 (7.0:7.2)
**Indicator H**	158.7	180.8 (180.7:180.9)	160.9	180.8 (180.7:180.9)	153.9	179.1 (178.9:179.3)	181.1	214.2 (214.1:214.4)	154.1	172.7 (172.6:172.8)
**Indicator I**	22.6	21.2 (21.1:21.3)	22.3	21.0 (20.9:21.1)	23.3	21.4 (21.3:21.6)	25.2	21.9 (21.7:22.2)	21.9	21.0 (20.9:21.1)
**Indicator J**	95.9	81.4 (81.3:81.5)	92.7	79.3 (79.2:79.4)	102.9	85.3 (85.2:85.4)	87.5	81.0 (80.9:81.2)	98.4	81.4 (81.3:81.5)
**Indicator K**	23.9	21.7 (21.6:21.8)	24.5	22.8 (22.7:22.9)	22.5	19.6 (19.5:19.8)	27.7	22.5 (22.2:22.7)	22.7	21.5 (21.4:21.6)

GI, Gastrointestinal; OR, Odds ratio; CI, Confidence interval

[a] Number at risk and pre-intervention rates estimated as mean over the 4 quarters prior to intervention at each site

[b] Rate per 1000 patients at risk

[c] Fitted rates are adjusted for calendar time and general practice

Note: The calendar time estimate for this analysis is based on a shorter time span (pre implementation only) compared with the main analysis. Therefore the fitted rates calculated here for all practices will differ slightly those shown in the main analysis (Table 4).
