# Supplementary material for: Scaling-up a pharmacist-led information technology intervention (PINCER) to reduce hazardous prescribing in general practices: Multiple interrupted time series study
Source: PLoS Med. 2022 Nov 16;19(11):e1004133. doi: 10.1371/journal.pmed.1004133 (PMC9718399; doi:10.1371/journal.pmed.1004133)

S7 Appendix. Plots for each Individual Indicator

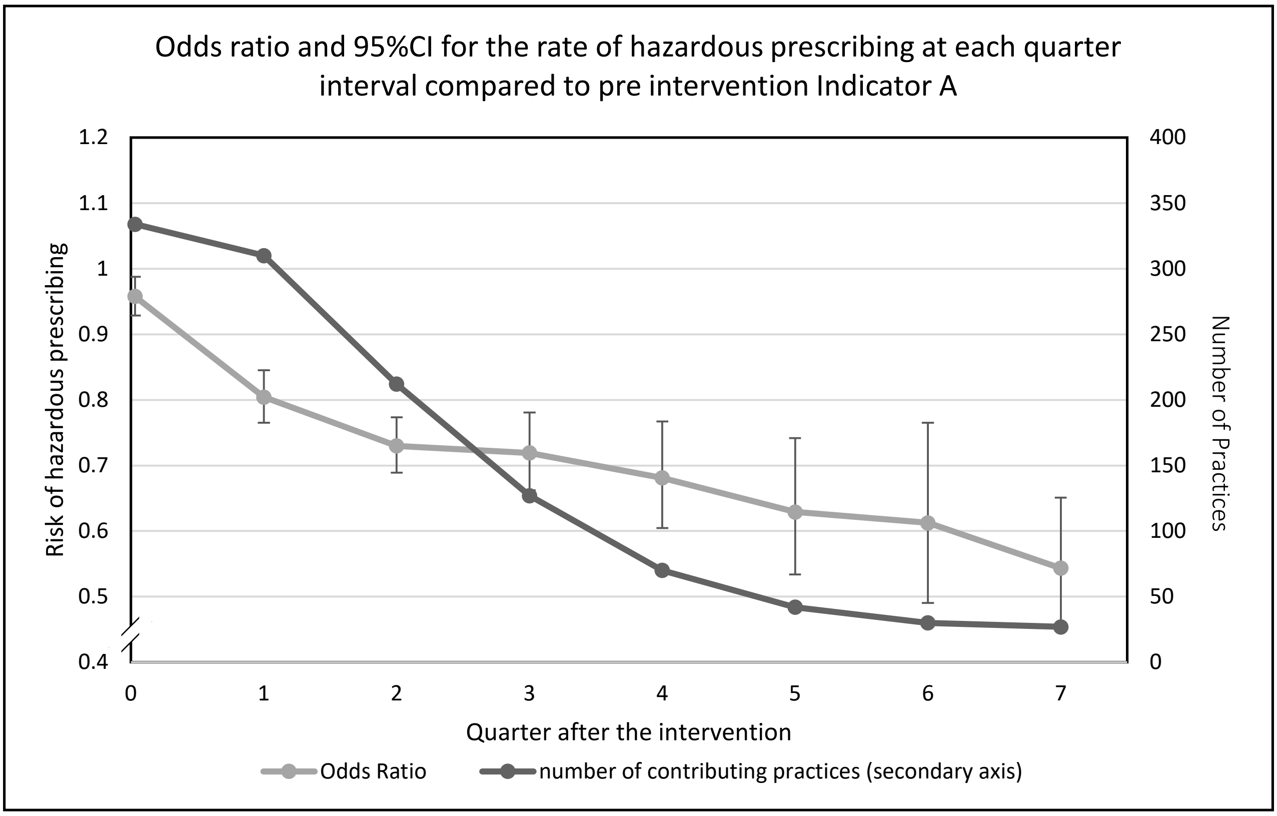

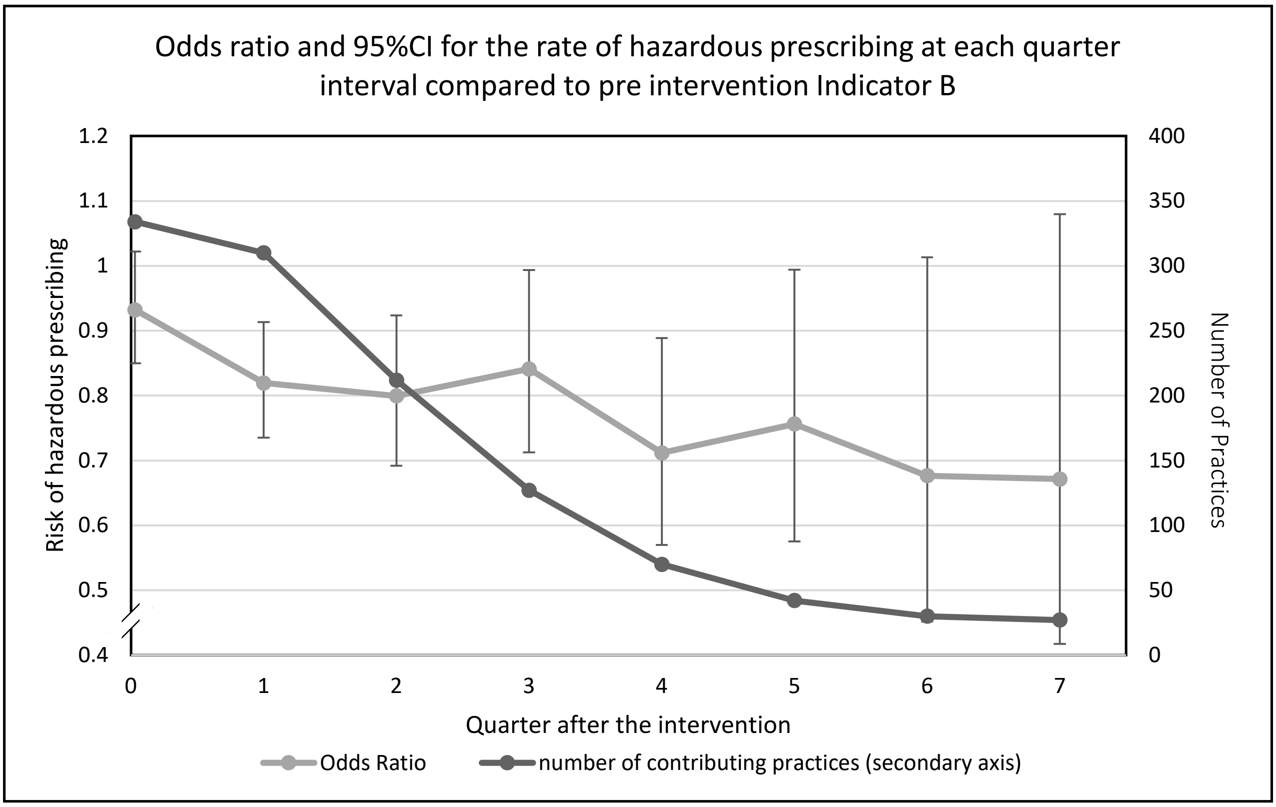

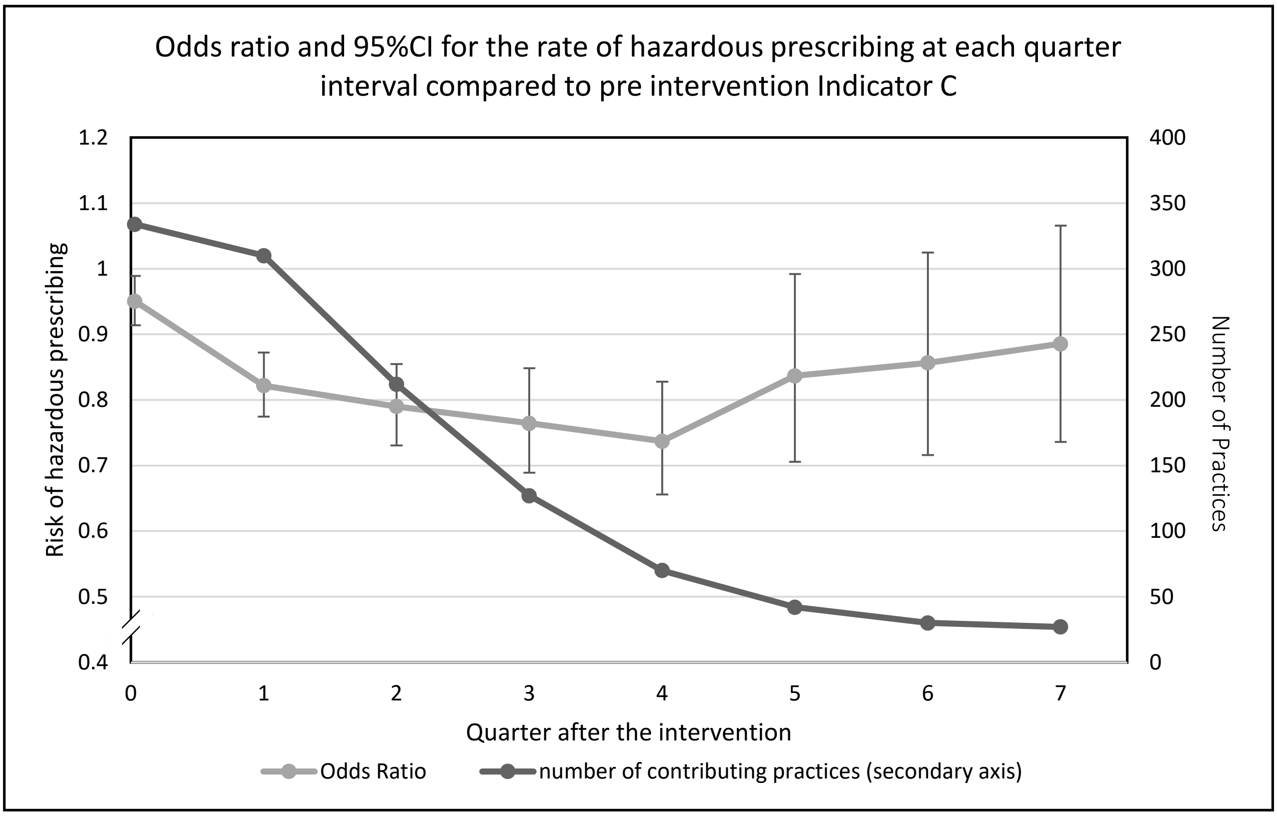
Odds ratio and 95%CI for the rate of hazardous prescribing at each quarter interval compared to pre intervention Indicator C
Risk of hazardous prescribing
Number of Practices
1.2
1.1
1
0.9
0.8
0.7
0.6
0.5
0.4
400
350
300
250
200
150
100
50
0
0
1
2
3
4
5
6
7
Quarter after the intervention
Odds Ratio
number of contributing practices (secondary axis)

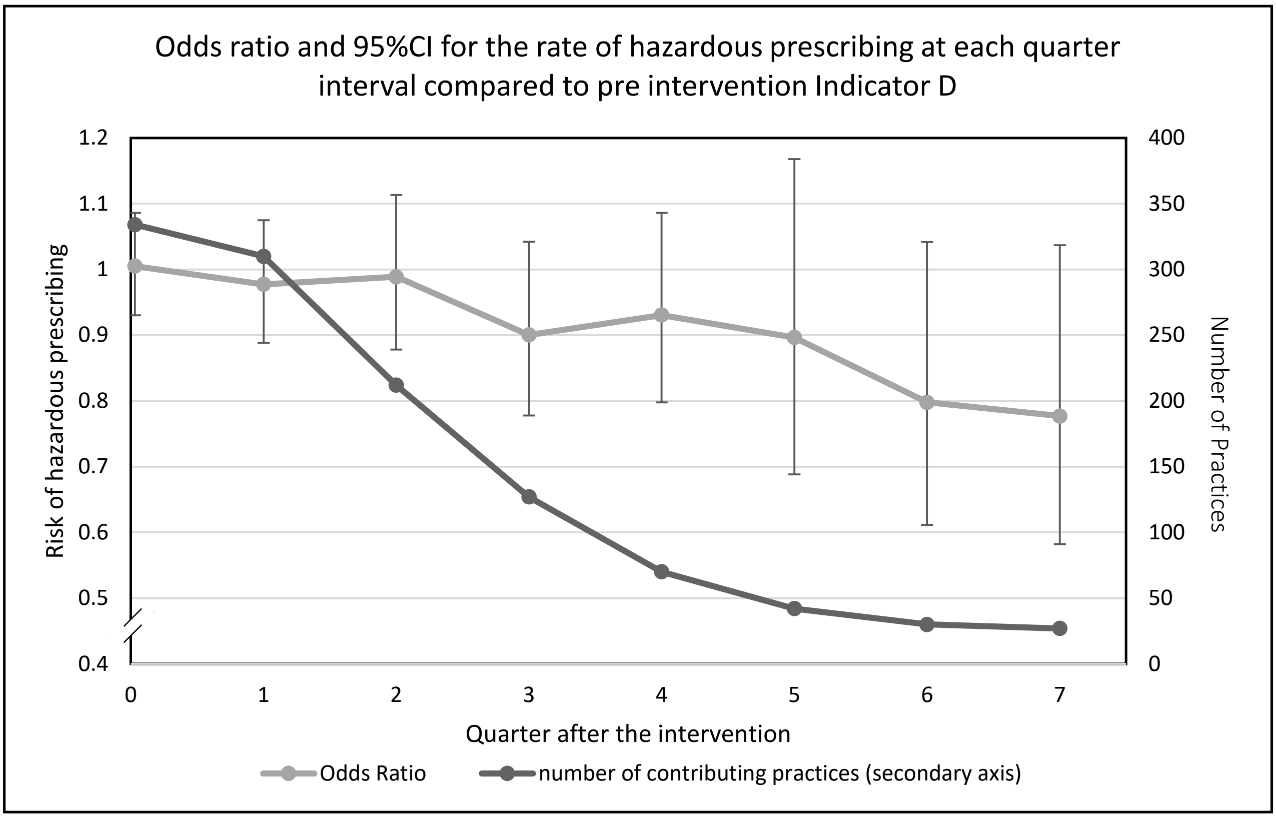
Odds ratio and 95%CI for the rate of hazardous prescribing at each quarter interval compared to pre intervention Indicator D
Risk of hazardous prescribing
Number of Practices
1.2
1.1
1
0.9
0.8
0.7
0.6
0.5
0.4
400
350
300
250
200
150
100
50
0
0
1
2
3
4
5
6
7
Quarter after the intervention
Odds Ratio
number of contributing practices (secondary axis)

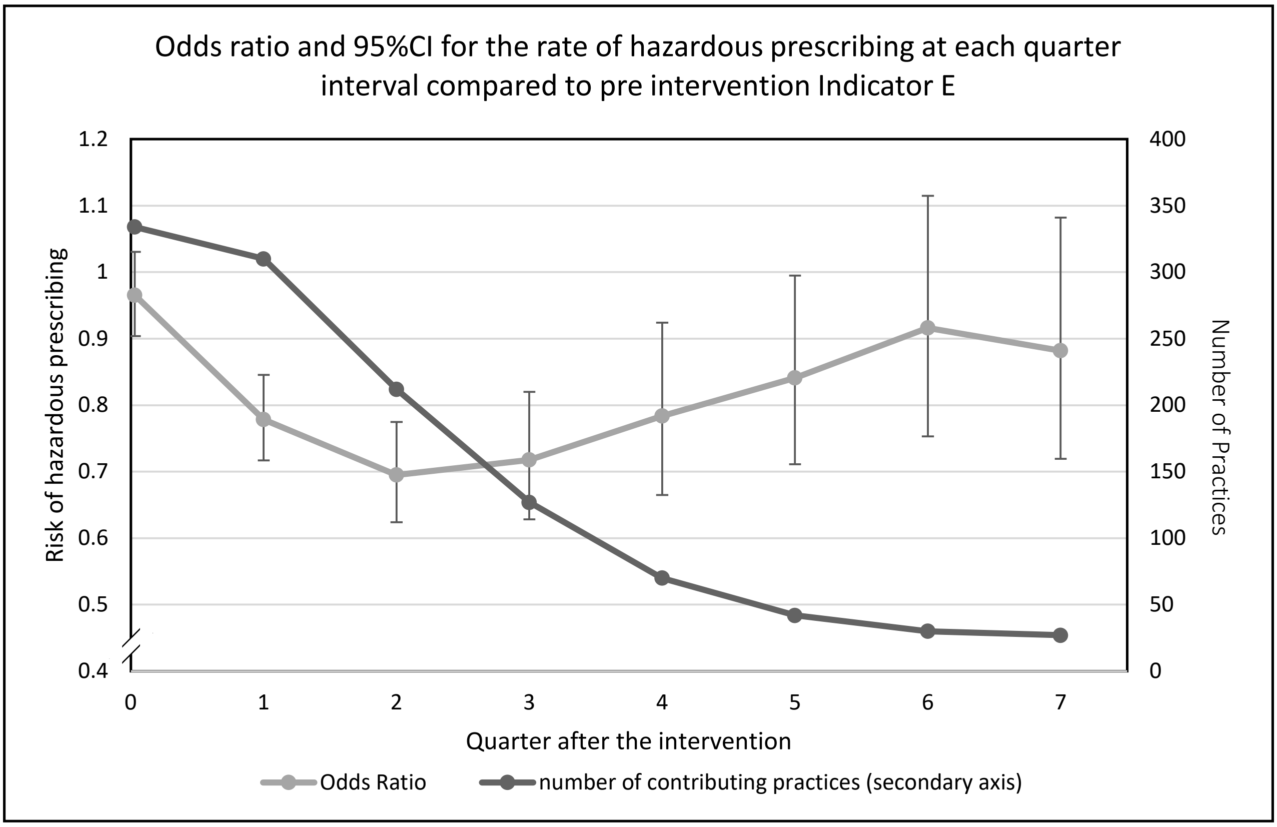

Odds ratio and 95%CI for the rate of hazardous prescribing at each quarter interval compared to pre intervention Indicator E
Risk of hazardous prescribing
Number of Practices
1.2
1.1
1
0.9
0.8
0.7
0.6
0.5
0.4
400
350
300
250
200
150
100
50
0
0 1 2 3 4 5 6 7
Quarter after the intervention
Odds Ratio
number of contributing practices (secondary axis)

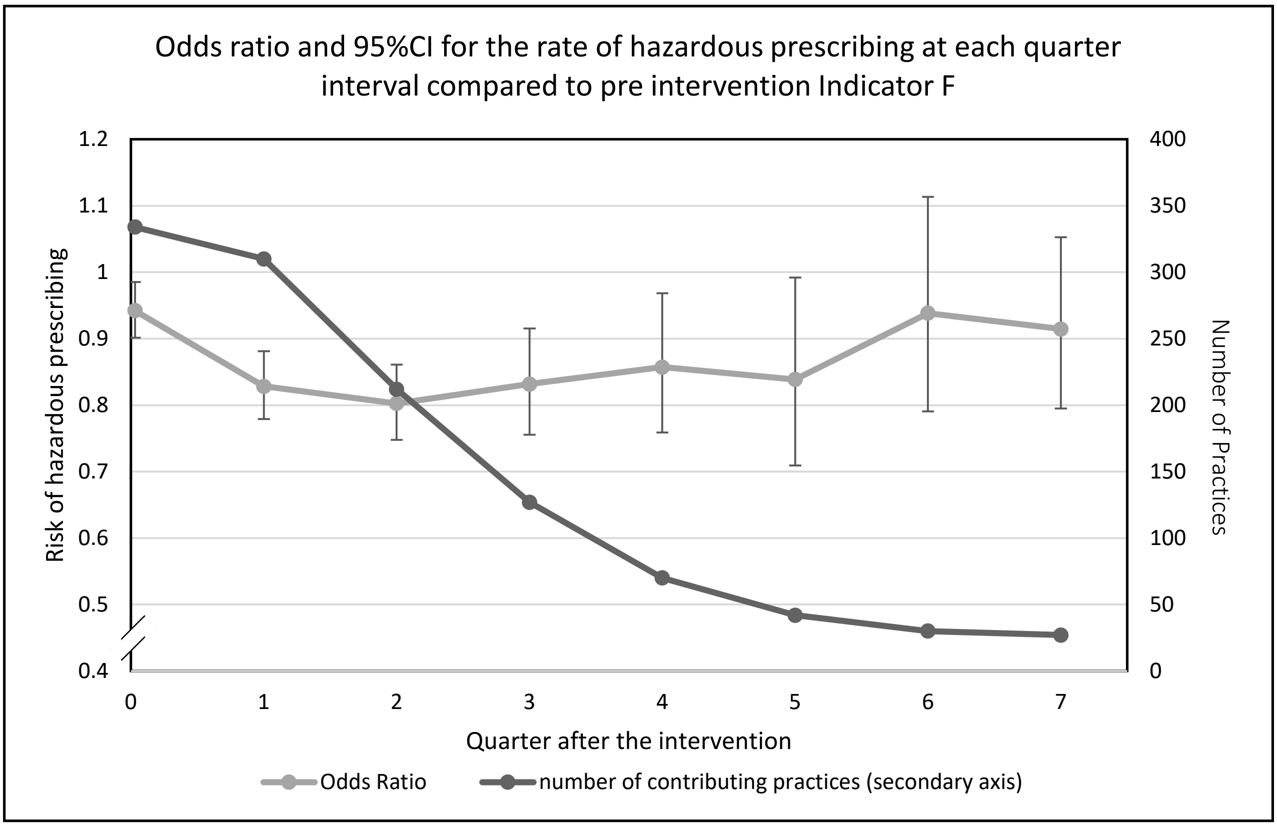

Odds ratio and 95%CI for the rate of hazardous prescribing at each quarter interval compared to pre intervention Indicator F
Risk of hazardous prescribing
Number of Practices
1.2
1.1
1
0.9
0.8
0.7
0.6
0.5
0.4
400
350
300
250
200
150
100
50
0
0 1 2 3 4 5 6 7
Quarter after the intervention
Odds Ratio
number of contributing practices (secondary axis)

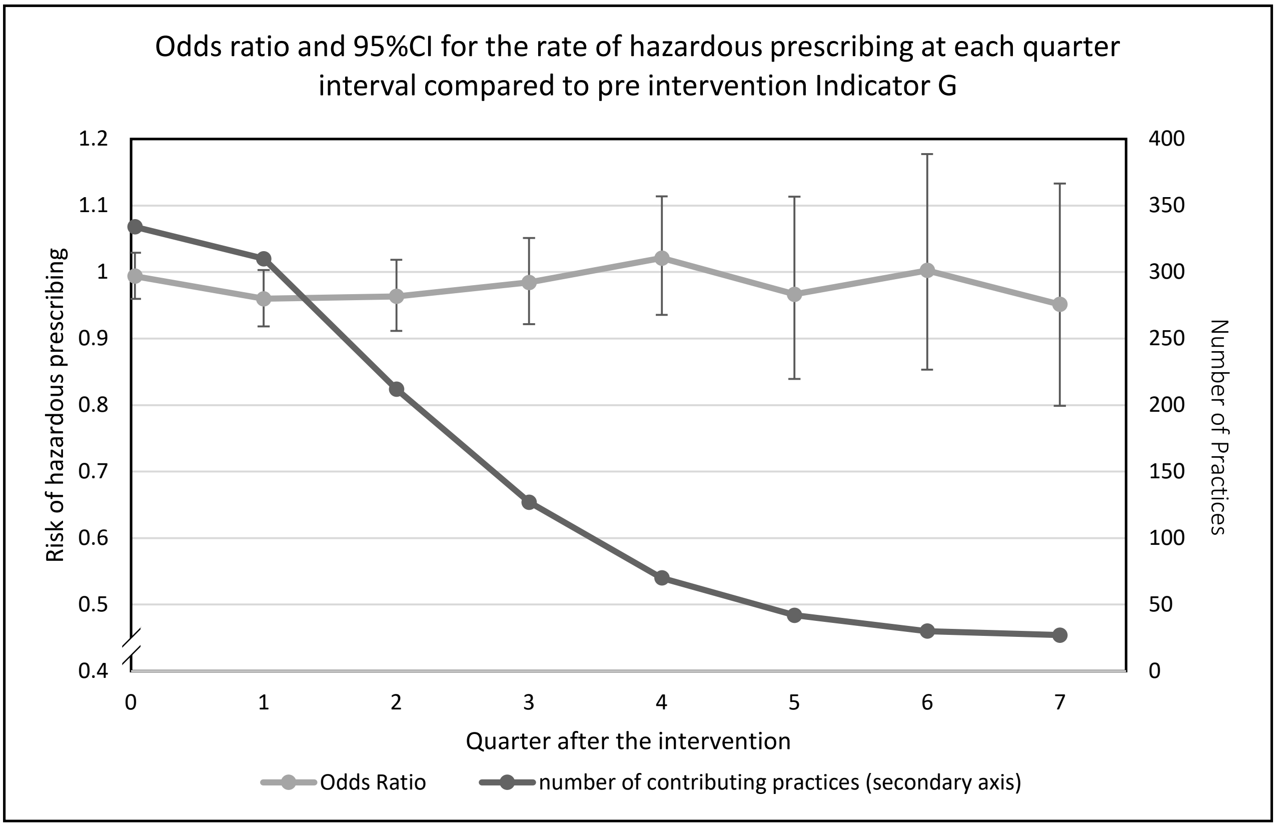

Odds ratio and 95%CI for the rate of hazardous prescribing at each quarter interval compared to pre intervention Indicator G
Risk of hazardous prescribing
Number of Practices
1.2
1.1
1
0.9
0.8
0.7
0.6
0.5
0.4
400
350
300
250
200
150
100
50
0
0
1
2
3
4
5
6
7
Quarter after the intervention
Odds Ratio
number of contributing practices (secondary axis)

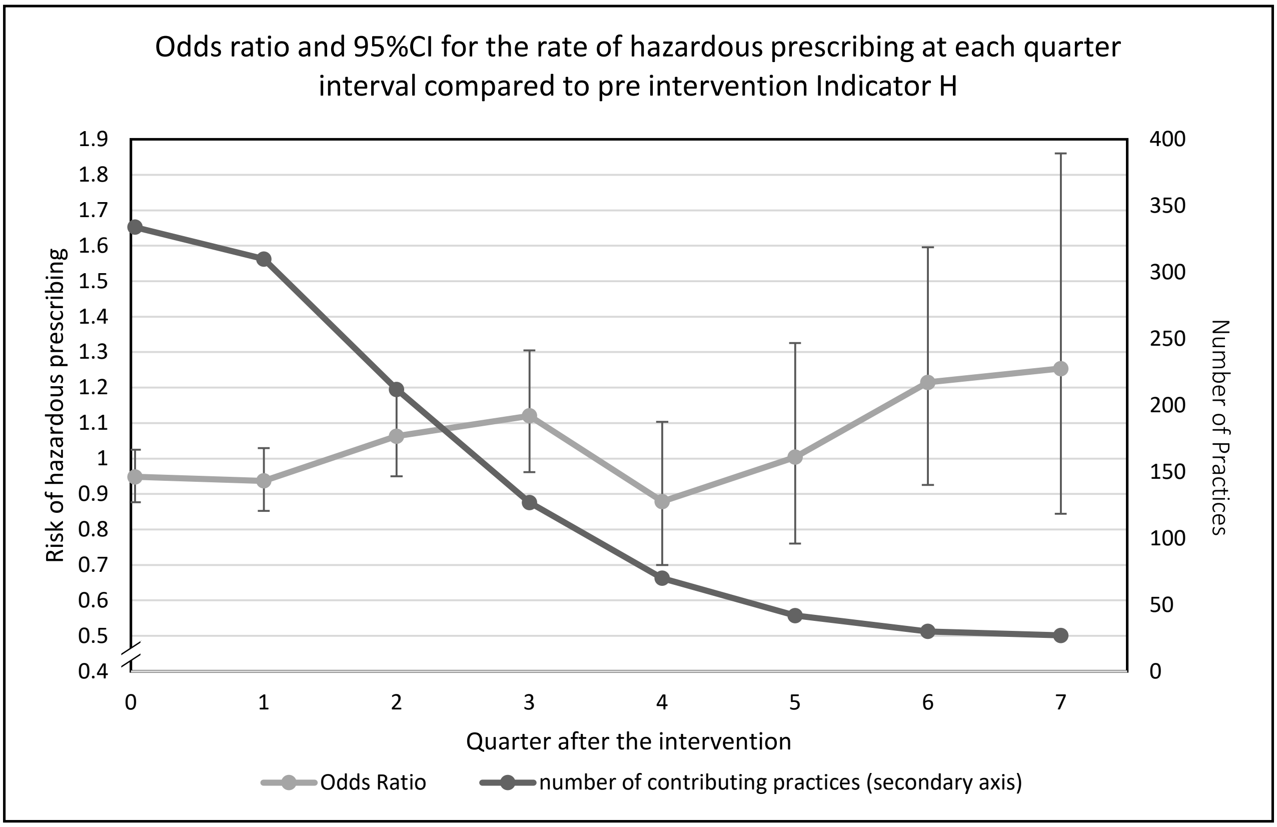

Odds ratio and 95%CI for the rate of hazardous prescribing at each quarter interval compared to pre intervention Indicator H
Risk of hazardous prescribing
Number of Practices
1.9
1.8
1.7
1.6
1.5
1.4
1.3
1.2
1.1
1
0.9
0.8
0.7
0.6
0.5
0.4
400
350
300
250
200
150
100
50
0
0
1
2
3
4
5
6
7
Quarter after the intervention
Odds Ratio
number of contributing practices (secondary axis)

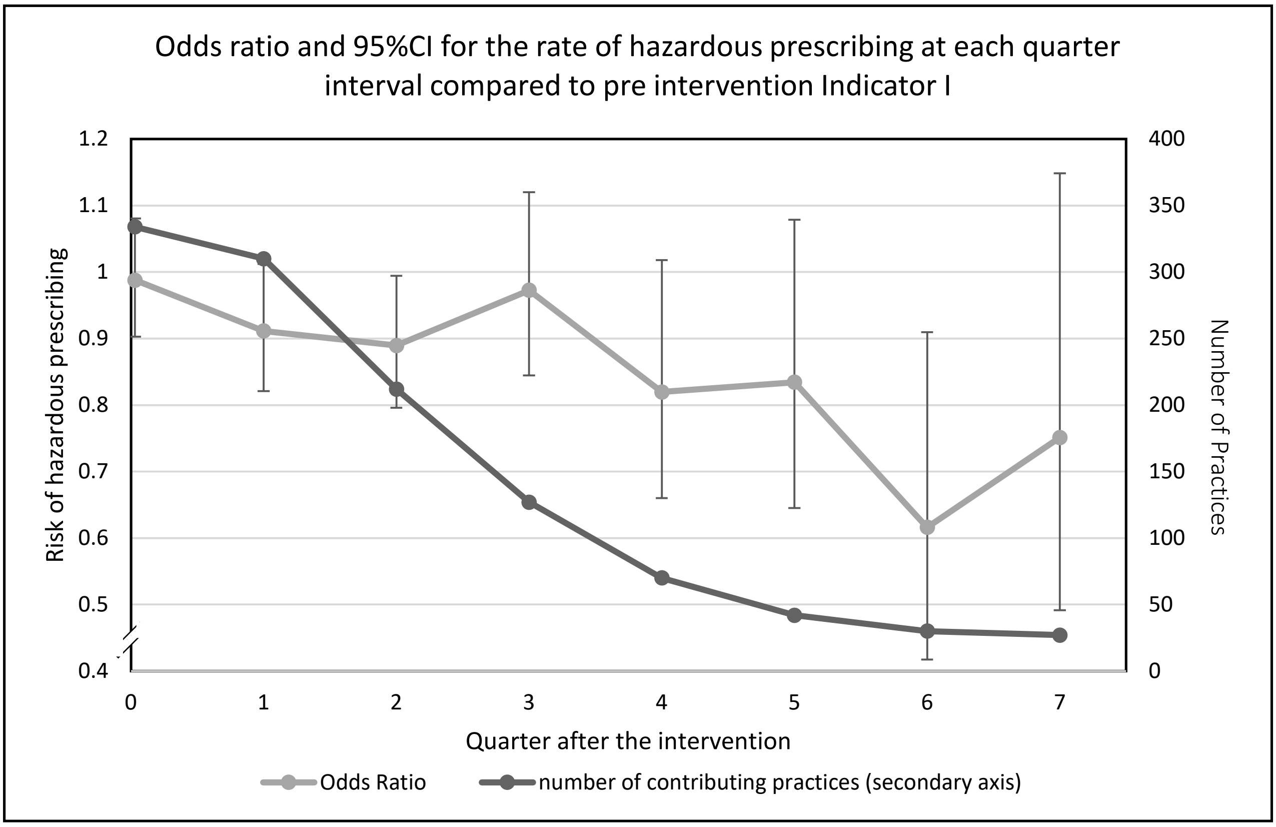

Note: the y axis has a larger range on this chart

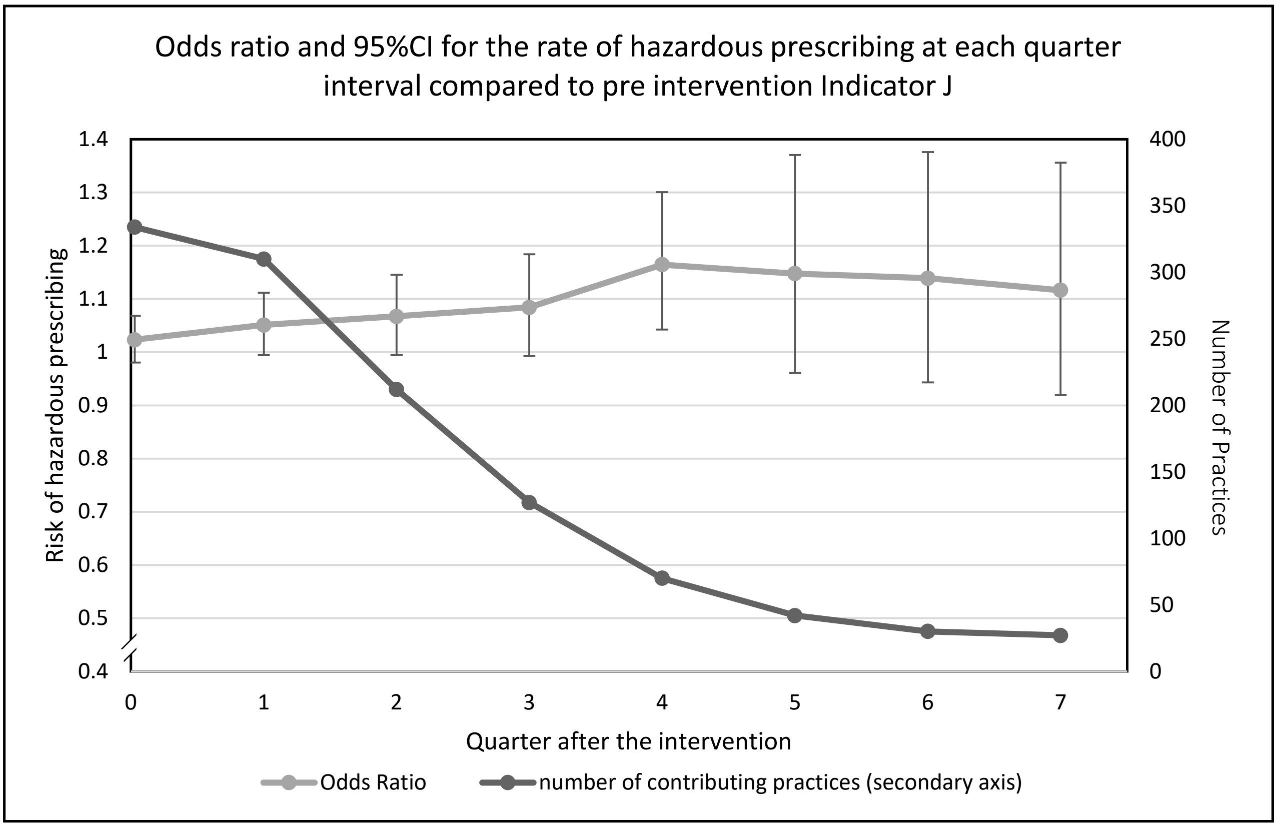

Note: the y axis has a larger range on this chart

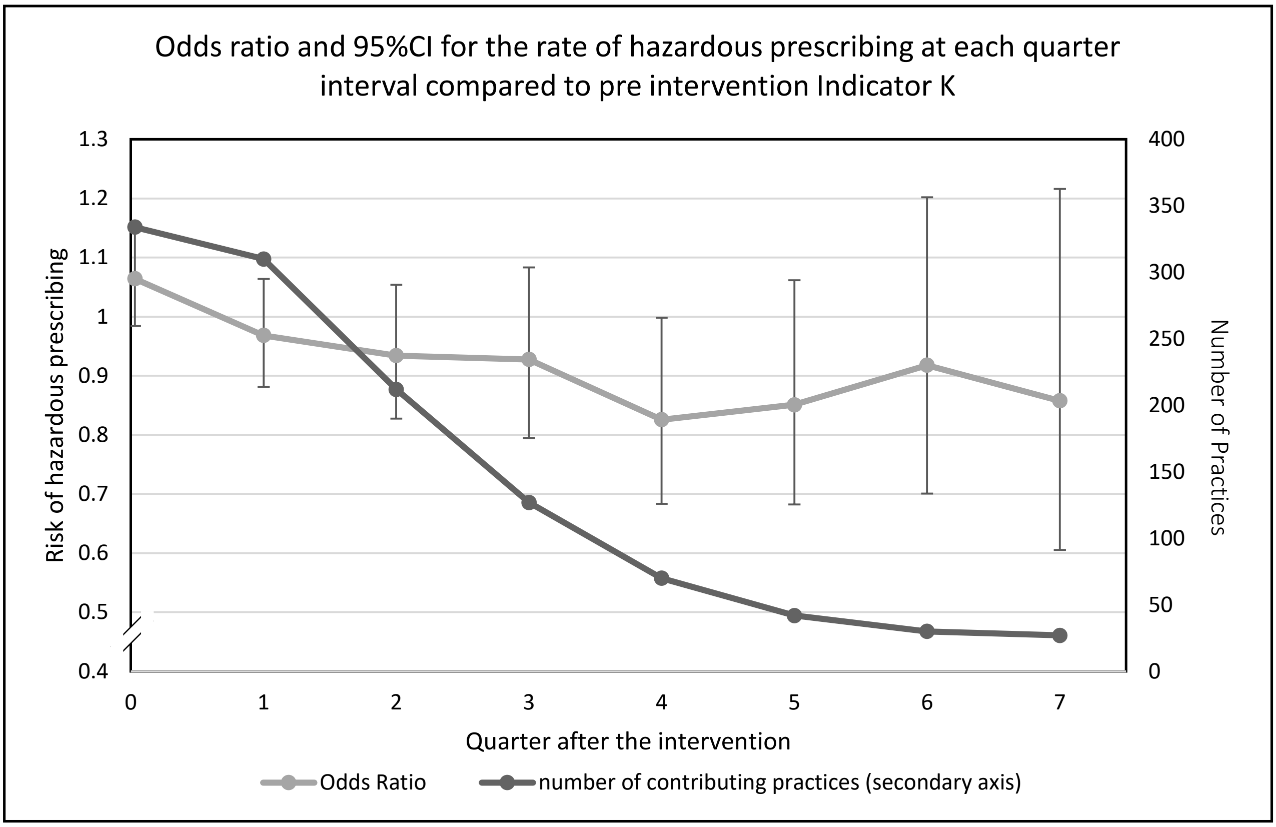

Supplement: S7 Appendix — (PDF) [file pmed.1004133.s007.pdf]
