# Supplementary material for: Scaling-up a pharmacist-led information technology intervention (PINCER) to reduce hazardous prescribing in general practices: Multiple interrupted time series study
Source: PLoS Med. 2022 Nov 16;19(11):e1004133. doi: 10.1371/journal.pmed.1004133 (PMC9718399; doi:10.1371/journal.pmed.1004133)
Supplement: Attachment — Submitted filename: PINCER HF paper reviewers comments 10 Aug 22.pdf [file pmed.1004133.s009.pdf]

comment reference number	reviewer/ section	Reviewers comments	Authors response	location of amendment - tracked version	location of amendment - clean version
1	Requests from the editors:	1) The Data Availability Statement (DAS) requires revision. For each data source used in your study:<br>a) If the data are freely or publicly available, note this and state the location of the data: within the paper, in Supporting Information files, or in a public repository (include the DOI or accession number).<br>b) If the data are owned by a third party but freely available upon request, please note this and state the owner of the data set and contact information for data requests (web or email address). Note that a study author cannot be the contact person for the data.<br>c) If the data are not freely available, please describe briefly the ethical, legal, or contractual restriction that prevents you from sharing it. Please also include an appropriate contact (web or email address) for inquiries (again, this cannot be a study author).	Thank you, we have removed the DAS from this location and instead will update it in the submission form.<br>Changed to:<br>'We are unable to share the practice level data as the data sharing agreement, provided to the practices involved, restricted the use of the data to PRIMIS, the Clinical Commissioning Group and members of the study team. It stated that data would not be shared with any person or organisation that does not appear in this list. For enquiries about the data, please contact 'sponsor@nottingham.ac.uk'.	removed line 23-24	n/a
2		2) Please remove the 'Funding," "competing interests" and "Data availability statement" after the title page. In the event of publication, this information will be published as metadata based on your responses to the submission form.	Removed as requested.	removed line 25-38	n/a
3		3) Abstract:<br>a) Please structure your abstract using the PLOS Medicine headings (Background, Methods and Findings, Conclusions). Please replace the subheading "Objectives" with "Background"<br>b) Please ensure that all numbers presented in the abstract are present and identical to numbers presented in the main manuscript text.<br>c) Please quantify the main results (with 95% CIs and p values).<br>d) Please provide the actual numbers of events for the outcomes (numerator and denominator), not just summary statistics or ORs.<br>e) In the last sentence of the Abstract Methods and Findings section, please describe the main limitation(s) of the study's methodology.	a) Thankyou- we have updated the Background header.<br>b) checked<br>c) Please see comment reference 8e.<br>d) We have added these numbers, but it has made the word count for the abstract a little higher<br>e) added a brief summary of the limmitations	a) line 37<br>d) line 64-68<br>e) line 79-82	a) line 31<br>d) line 48-52<br>e) line 63-66
4		4) c - At this stage, we ask that you reformat your non-technical Author Summary. The Author Summary should immediately follow the Abstract in your revised manuscript. This text is subject to editorial change and should be distinct from the scientific abstract. The summary should be accessible to a wide audience that includes both scientists and non-scientists. Please see our author guidelines for more information: https://journals.plos.org/plosmedicine/s/revising-your-manuscript#loc-author-summary.	Thank you. We have added this as a new section.	line 84-110	line 68-94
5		5) Your study is observational and therefore causality cannot be inferred. In the abstract and discussion, please remove language that implies causality, such as "reduced hazardous prescribing" or similar. Refer to associations instead.	Thank you. We have changed the wording in abstract and discussion	lines 68, 426, 439, 638	lines 52, 376, 383, 512
6		6) Please add the following statement, or similar, to the Methods: "This study is reported as per the Strengthening the Reporting of Observational Studies in Epidemiology (STROBE) guideline (S1 Checklist)."	Thank you, this has been added and the checklist completed.<br>Checklists - STROBE (strobe-statement.org)	line 280	line 253
7		7) When completing the STROBE checklist, please use section and paragraph numbers, rather than page numbers.	Thank you, this has been added and the checklist completed.	S3 Appendix	S3 Appendix
8		8) In the methods and results please address the following:<br>a) In the main text, please provide the actual numbers (numerator and denominator) for the outcomes, not just summary statistics or ORs.<br>b) Please present both numerators and denominators for rates, in the Table 4<br>c) Please provide both adjusted analyses and unadjusted analyses, where appropriate<br>d) In the text, please provide p values in addition to 95% CIs when describing numeral results<br>e) When a p value is given, please specify the statistical test used to determine it.	a) We have added the numerators and denominators to the results section.<br>b) and c) The numerators and denominators and the unadjusted rates have been added to table 4.<br>d) and e) We have adopted an effect-size approach to the analysis and presentation and deliberately avoided the use of P-values. This is standard practice in many epiidemiology journals and has been strongly recommended in most major journals in recent years (see for example editorials in Nature)	a) lines 371-374 and 377-381<br>b),c) Table 4	a)lines 343-346 and 349-353<br>b),c) Table 4
9		9) In Figure 2 and S4, please show the Y axis beginning at zero. If this is not possible, please show a break in the axis.	Thank you. A break in the axis has been added.	Figure 2, Appeddix S7	Figure2, Appeddix S7
10		10) In the tables, please define all abbreviations e.g., CCG, Q, GI	Thank you, these are now added.	line 388,<br>S1 Appendix<br>S5 Appendix<br>S6 Appendix	line 356.<br>S1 Appendix<br>S5 Appendix<br>S6 Appendi
11		11) In the table S3 footnotes, please define what Indicator A, B, C… stand for.	Thank you, these are now added.	S5 Appendix	S5 Appendix
12		12) Please remove subheadings in the Discussion section, and present and organize the Discussion as follows: a short, clear summary of the article's findings; what the study adds to existing research and where and why the results may differ from previous research; strengths and limitations of the study; implications and next steps for research, clinical practice, and/or public policy; one-paragraph conclusion.	We have done this, thanks.	lines 422- 641	lines 373- 515
13		13) References:<br>a) Please ensure that journal name abbreviations consistently match those found in the National Center for Biotechnology Information (NCBI) databases. https://journals.plos.org/plosmedicine/s/submission-guidelines#loc-references.<br>b) Please include access dates for all weblinks and ensure that all weblinks are current and accessible, e.g. ref #4	a) Thank you, all updated.<br>b) Checked and added	lines 643-692	lines 516- 564

14	Comments from reviewer 1:	Issue 1:<br>The authors state in line 143 that data was extracted retrospectively. That up to 16 quarters were extracted (Dec 2013 to Aug 2017). Then in 147 they state that not all practice contributed 16 quarters of data and that for most data was extracted in Oct or Nov 2017.<br>The number of practices included in the analysis changes with time and this is summarised in table S2. The quarter before the intervention period (quarter -1) there are 341 practices included and for the 7 quarters preceding this there are between 341 and 343 practices. For the period of time at the start of the intervention there were 343 practices, then 310 in the following quarter (quarter +1) and 212 in the next (quarter +2). By quarter +4 there were 70 practices and by quarter +7 there are only 27 practices.<br>This means the primary statistical analysis of improvement at 6 months (quarters +2) was based on approximately two thirds of practices and the secondary analysis at 12 months (quarters +4) in one fifth of practices.<br>It could be argued that the paper is redrafted to only include the 212 practices with sufficient data to analyse the primary end point with a sensitivity analysis conducted with the subset of practice (70) with sufficient data to analyse the secondary end point. The other 131 practices (343 less 212) are contributing data to the baseline analysis but not the post-intervention period and estimation of intervention effect size.<br>As a minimum the number of GP practices contributing data to the primary and secondary end points should be made clearer at the start of the results section (currently it is at the end - line 284). Adding the number of practices contributing data to each quarter to the Figure 2 and S4 plots would help make this clearer.	Thank you, this is helpful. We have moved the description of the number of practices up to earlier in the results section, and added the number of contributing practices to the charts (Figure 2 and S4) on a secondary axis.	line 284<br>Figure 2<br>S7 Appendix	line 312<br>Figure 2<br>S7 Appendix
15		Issue 2:<br>The analysis method is described in the title as an interrupted time series analysis (ITSA).<br>The statistical methodology estimates a single pre-implementation base rate (mean of the 4 quarters immediately pre-intervention) with post-intervention values estimated at 6 months and 12 months from temporal trends. However, this appears to mean that pre-intervention trends are not used to estimate the effect size from the counterfactual trend line and that the temporal trends estimate in the post intervention period for the primary outcome are based on two values (quarter +1 and quarter +2). The minimum number recommended for Cochrane systematic reviews of ITSA is three pre and three post intervention. It would appear that according to Cochrane criteria this study would not be included in an Effective Practice and Organisation of Care (EPOC) review<br>https://epoc.cochrane.org/sites/epoc.cochrane.org/files/public/uploads/inttime.pdf<br>A review of the statistical methodology by a statistician would be useful to assess the appropriateness of the statistical method and the use of the description "interrupted time series study".	We agree that this is not a classic ITSA and we have not used this terminology. It can be thought of as a multiple interrupted time series *design* as in the title. The model does include a linear temporal effect over the pre-intervention period and estimates a pre-intervention effect from this and the post-intervention effects are the differences from this extrapolated trend. However we were a priori not expecting a sustained effect from the single intervention so we did not use the classic linear post-intervention effect (step plus change in slope) but rather parameterised the model to allow for a non-linear intervention effect. For the record there were more than 10 pre-implementation data points for most centres. Finally we do note that one of the authors is a highly experienced staistician and that the methodology was pre-specified and reviewed prior to the start of the analysis.<br>To make this more explicit we have added the following text to the beginning of the statistical methods section of the paper:<br>"Although we used a multiple interrupted time series design, we did not conduct a classic interrupted time series analysis. Instead, the model we used includes a linear temporal effect over the pre-intervention period and estimates a pre-intervention effect from this; the post-intervention effects are the differences from this extrapolated trend. We were not expecting a sustained effect from the intervention, so we did not use the classic linear post-intervention effect (step plus change in slope) but rather parameterised the model to allow for a non-linear intervention effect".		
16		Other minor issues:<br>1. The table footnotes in Table S3 are missing (a,b,c,d are not defined)<br>2. CIs in Table S3 12months OR (last column) have additional spaces which do not exist for the other CIs in Table 4 or Table S3	1) added (see point 11).<br>2) Thank you, We also noted the same spaces for the 6 month data, so have changed both to delete the spaces.	S5 Appendix (was S3)	S5 Appendix (was S3)
17	Comments from reviewer 2:	Firstly, whilst almost 350 practices were recruited, data from only 212 were analysed at 6 months and only 70 at 12 months. The authors have included a sensitivity analysis to determine whether including data from practices where they have full data at 6 months produces the same outcomes as the partial data analysis and have confirmed that this is the case. However, they have not provided any explanation as to why the drop out rate is so high nor have they considered that those practices with full-data at 6 months may be more engaged and therefore have improved outcomes compared to those who dropped out? What are the differences between the characteristics of the practices that data was obtained at 6 and 12 months compared to those that started the initiative? Was the difference in engagement due to level of pharmacist support between practices? No information is provided in the manuscript regarding this detail. Particularly given that the PINCER study demonstrated better results with pharmacist support this is important.	Applogies, but we did not make it clear that practices did not drop out of the study. Due to the fact that some practices uploaded their own data to CHART Online, there were some gaps in the data in the post-intervention quarters, including quarter 0. All practices had access to their own PINCER data; it is the data that was shared with the research team that wasn't complete for all practices.<br>We have added the characteristics of the practices with 6 months (n=212) and 12 months (n=70) follow up data for comparison to Table 2 and found no consistent differences. We have also added the following text to the Results section "The reduction in the number of practices at folow up, was not believed to be related to the practices engagement with PINCER, but instead whether they uploaded the data to CHART Online, to be shared with the research team."	Table 2, lines 329<br><br>306-308	Table 2, lines 301<br><br>279-281

18		Figure 2 shows that the 95% confidence intervals post intervention (for GI and all outcomes) are much greater than pre-intervention. Why is this? Does this suggest that the intervention becomes less effective over time for some practices? Why? - is the presence or otherwise of a pharmacist contributing to this variability or are those practices who are rated as poor/ needs improvement by the CQC less likely to be following up on the recommendations with time or is there another explanation?	The narrower confidence interval pre-intervention reflects the fact that there were many more periods contributing to the estimates for this period. A downside of the model we (pre)specified is that there is very little "borrowing" of strength across the post-intervention period - each time point is close to a stand-alone comparison with the pre-intervention trend (we were fortunate here in having a large dataset with the power to allow this analysis and to fully model the post-intervention time course).<br>We have added the text:<br>"The 95% confidence intervals are much larger post intervention than pre intervention. This is due to the number pre intervention quarters that were combined to contributing to the pre intervention data point, where only single quarters contributed to each of the post implementation data points."	lines 397-400	lines 364-367
19		The authors also fail to comment on why the prescribing of long acting beta-2 agonists to patients with asthma who were not receiving inhaled steroids was so high nor why it failed to improve at all with the intervention.	On reviewing our manuscript we have not commented on failures to improve some of the other outcomes. We have addressed this as follows: In the results section we have added: "No reduction in the rate of hazardous prescribing was found for indicators associated with asthma (G and H) or stroke (J)".<br><br>In the discussion section we have added: "In contrast, no reduction in the rate of hazardous prescribing at 6 months post-intervention was found for indicators D (NSAID and anticoagulant prescribed concurrently), G and H (asthma patients prescribed non-selective beta-blockers and asthma patients prescribed a long-acting beta-2 agonist inhaler but not also prescribed an inhaled corticosteroid), and J (dementia patients prescribed antipsychotics). We can only speculate on the reasons for this, with the most likely one being that pharmacists found it more difficult to make these changes."	Results-lines 402-403<br><br>Discussion-lines 444-449	Results-lines 368-369<br><br>Discussion-lines 387-392
20		Please clarify why outcome J is labelled stroke - the prescription of antipsychotics to patients with dementia without a diagnostic code of psychosis.	The indicator J (Patients aged ≥65 years with a Read code for dementia but no Read code for psychosis prescribed antipsychotic drugs for >6weeks) is related to the serious harm outcome of a stroke. This is consistent with the other indicators, referred to by the serious harm outcome that the intervention related to that indicator is trying to prevent, such as GI bleed or an exacerbation of asthma.<br><br>To make this clearer in the paper, we we have added the following sentence to the section 'Outcome measures' :<br>These 11 indicators are associated with the following serious harm outcomes; gastrointestinal bleeding, asthma, heart failure, stroke and acute kidney injury.	lines 207-208	lines 189-190
21	Comments from reviewer 3:	Line 118: I appreciate these indicators are from the original PINCER trial - but could you provide a reference to how these indicators were informed.	We have added the following text to the introduction section:<br>"Therefore, we developed a set of prescribing safety indicators[REF Spencer] to identify patients exposed to medication errors in general practice, and the PINCER intervention is designed to ameliorate risk from the most common and important of these errors. "<br><br>Spencer R, Bell B, Avery AJ et al. Identification of an updated set of prescribing-safety indicators for GPs. BJGP 2014;64(621):e181-e190. https://doi.org/10.3399/bjgp14X677806	lines 144-146	lines 128-130

22	Line 123: Please provide more information regarding the pharmacists involved in the intervention. Were they co-located in the general practice? How many hours per week were they working in the general practice? What experience/training did they have prior to the study? Were they experienced general practice pharmacists? Were they prescribers?	This was a pragmatic implementation study. The intervention was delivered by the pharmacists employed in each of the CCG Medicines Optimisation teams. Training on the PINCER intervention (half-day) and CHART software (half-day) was successfully delivered to members of the Medicines Management Teams in all 12 CCGs that implemented the PINCER intervention.<br>In total, 118 pharmacists/pharmacy technicians and 18 other staff, including GP practice staff and CCG MMT members, received PINCER training prior to PINCER implementation.<br><br>We have added the following description:<br>The East Midlands rollout was a pragmatic implementation study and the PINCER intervention was delivered by the pharmacists employed in each of the CCG Medicines Optimisation teams. Training on the PINCER intervention and CHART software was delivered to members of the Medicines Management Teams in all 12 CCGs that implemented the PINCER intervention.	lines 180-183	lines 162-165
23	Line 126: How often did these discussions take place, where and when did they take place?	It was recommended during training that a practice feedback session should be held straight after running the searches and again at regular intervals to follow-up on tasks assigned to practice team members and report on improvements made in order to complete the QI cycle and bring about change<br>We have added the following description:<br>"These feedback sessions were to be held straight after running the searches and then at regular intervals."	lines 170-171	lines 153-154
24	Line 127: Please provide an example of these educational materials.	We have added the following description:<br>"The educational materials included training on Root Cause Analysis using well established techniques such as the Fishbone diagram and the 5 why's."	lines 171-172	lines 151-155
25	Line 128: Was the action plan retained in the general practice?	Yes, the action plans were retained within the practice.<br>We have added the following description:<br>•Agree an action plan, retained within the practice...	line 173	line 156
26	Line 130-131: This section states that pharmacists supported by technicians implemented the action plan. Were these pharmacists/technicians in the community setting? If so, where was the action plan retained? How did the pharmacist actually "support general practice staff" in implementing the action plan?	The majority of the pharmacists/technicians involved in the implementation of PINCER were employed by the CCG and routinely worked in general practices within their localities. A small number of pharmacists involved in the implementation of PINCER were directly employed by the practice. We do not have full details of how exactly the pharmacists supported the practices (as this was a pragmatic study and we did not undertake a process evaluation) but we have added the following text:<br><br>"Third, the pharmacists (sometimes supported by pharmacy technicians) work with, and support, general practice staff to implement the agreed action plan [original text]; sometimes making the necessary changes themselves [added text]. "<br><br>The East Midlands rollout was a pragmatic implementation study, and the PINCER intervention was delivered by the pharmacists employed in each of the CCG Medicines Optimisation teams. Training on the PINCER intervention and CHART software was delivered to members of the Medicines Management Teams in all 12 CCGs that implemented the PINCER intervention. The time that the pharmacists varied by CCG, depending on the resourcing level of each of the CCG Medicines Optimisation Team. For example, we know some localities were very well resourced in terms of numbers of CCG pharmacists whereas other localities had very little CCG pharmacist resource".	lines 177-178<br><br>lines 180-186	lines 159-160<br><br>lines 162-168
27	Line 146: Although the authors have referenced the original paper, it would be good to have an idea of what this processing included.	We have added the following text:<br>"This included collation of numerators and denominators for each of the indicators for each of the time points, and calculation of composuite indicators"	lines 202-203	lines 184-185
28	Line 156-157: "For most practices, retrospective data extraction was carried out during October 2017 and November 2017." Does this mean that practices only carried out data extraction at two time points?	We have reworded this to make it clearer- see point 29.	see comment 29	see comment 30
29	Line 149-198: My main comment about this section is that it is quite hard to follow when data were collected, extracted, action plans were implemented etc. Would suggest revising how this is reported and perhaps include a timeline or the similar.	We have reworded the Outcome Measures section to make it clearer. There is a timeline for when the practices implemented PINCER in table S1.	lines 206-218	lines 187-197
30	Line 166: Why were the gastro-intestinal indicators singled out as a group?	We have added the text:<br>"The gastrointestinal indicators were singled out as a group because there was a relatively large number of these, and other studies have suggested these may be particularly sensitive to intervention [reference DQIP (7) and SMASH (8)]".	lines 232-234	lines 207-209
31	Line 287: "We had complete data for all 343 practices." What does this mean? Does it mean that data were collected for all practices at all quarters? This links back to my comment that the timeline of data collection and analysis is difficult to follow.	Removed the word 'complete'.	line 304	line 277

32		Line 314: "The level of uptake of the intervention was very high with 94% (370/393) of eligible practices in the East Midlands of England participating in the implementation." In order to be counted as having participated, did the practice only have to submit one set of data at one quarter, i.e. they didn't have to continuously submit data at all quarters?	We have added to the results section:<br>"To be included, a practice must have ran the intervention and uploaded at least one quarter of data."	line 302	line 275
33		Line 322: What were these pragmatic reasons?	We have added the following text to the Strengths and limmitations section:<br><br>"Originally, we had planned to roll the intervention out using a randomised stepped-wedge design. However, it became clear that CCGs needed to include the rollout of PINCER in their work plans (which are set prior to each NHS financial year) and in some cases, the Medicines Optimisation Teams had to submit a business case to their CCG to enable them to re-purpose their pharmacist resource. This made the process of randomisation impossible."	line 511	line 441
34		Line 323: What were these "secular trends"?	The end of this sentence says 'the trend towards safer prescribing over time'<br>We have updated this sentence to explain how we have accounted fo the secular trend; "We did, however, take account of secular trends towards safer prescribing over time, by including the calendar reference date in the models".	lines 517-518	lines 445-446
35		Line 389-393: Although HCPs have been trained in PINCER, do the authors have any idea as to how much it is actually used in day-to-day practice?	We have stated that the intervention has been rolled out to over 2,800 general practices, which means that those practices are likely to have delivered the intervention at least once. We do not have information on how much it is used in day-to-day practice, although all general practices in England have been encouraged to adopt the PINCER approach as part of an incentive scheme in operation in 2022-23.<br>We have added this to the revised text: "For exmple, in 2022-23 all general practices in England have been incentivised to use the PINCER approach with pharmacists undertaking structured medication reviews for patients identified from PINCER indicators[Reference].<br>The reference is: Network Contract Directed Enhanced Service. Investment and Impact Fund 2022/23: Updated Guidance. Available at:<br>https://www.england.nhs.uk/wp-content/uploads/2022/03/B1357-investment-and-impact-fund-2022-23-updated-guidance-march-2022.pdf [accessed 15 July 2022]	lines 622-624	lines 499-501
36		Line 396-399: Can the authors better explain the relationship between pharmacists being employed in general practice and increased use of PINCER - is use of PINCER an explicit part of their job description?	It wasn't an explicit part of their job description, but the implementation of PINCER was written into the workplans of each of the CCG Medicines Optimisation Teams and in some instances, the CCGs incentivised the rollout of PINCER.<br><br>We have added the following text: "Pharmacists in general practice in England undertake a range of activities aimed at medicines optimisation and while delivery of the PINCER intervention is not necessarily an explicit part of the job description, there is ongoing policy drive towards the use of this approach". This is followed by the text described in the response to the previous comment: about the incentivisation for 2022/23 to use the PINCER approach.	lines 619-622	lines 496-499
37	Comments from reviewer 4: Overall	Three main concerns that the authors should address prior to publication:<br>-The way that the indicators on GI risk are specified, desirable changes in prescribing (i.e. initiation of gastroprotection) would lead to reductions in the denominator, which makes changes difficult to interpret. As a minimum, the authors should provide numerator and denominator data for all data points<br>-There has been substantial loss to follow up and the potential for bias resulting from this should be explored further by comparison of baseline data for those with and without loss to follow up<br>-The analysis methods appear pragmatic but their accounting for pre-intervention trends seems to fall short of conventional segmented regression of time series data. As a minimum, the authors should provide preintervention trends of rates of hazardous prescribing for all indicators	1.Thankyou. We have added numerators and denominators to the data points. (See coment 8 above).<br>2.We have added the practices who were included at 6 months and 12 months post intervention to table 2 to compare the characterostics of the practice. Please also see comment 42 about sensitivity analyses. We have also compared the raw and adjusted pre implementation rates for those practices with up to 6 months and up to 12 months follow up (Table S4). The rates for each group of practices were found to be similar. See comment 53.<br>3. The model employed here is essentially the same as the conventional segmented regression, but relaxing the constraint that the treatment effects confrom to a linear change (slope plus intercept). Please see our response to comment 15.<br>Figure 1 shows the unadjusted pre intervention trend the the composite and compoite GI indicators aligned by calendar quarter.	1) 371-374 and 377-381<br>2) Table 2	1)343-346 and 349-353<br>2) Table 2
38	Methods	1. "Third, the pharmacists (sometimes supported by pharmacy technicians) work with, and support, general practice staff to implement the agreed action plan." There is insufficient detail to understand what the pharmacist contribution comprised. Please, provide more detail as to the pharmacist time dedicated to PINCER work as well as the nature of their "support" to practice staff, e.g.<br>- Were the pharmacists present in the practices over the whole implementation period?<br>- If not, how long were they present? If so, what was the full time equivalent they dedicated to PINCER work?<br>- Did the pharmacists provide input into individual patient care?<br>- Did they access individual patient records?<br>- Did pharmacists see patients?<br>- Did pharmacists only advise on or help amend processes at practice level?	As this was a pragmatic study, this will have varied depending on the resourcing level of each of the CCG Medicines Optimisation Teams. For example, we know some localities were very well resourced in terms of numbers of CCG pharmacists whereas other localities had very little CCG pharmacist resource.<br><br>W have added the following explanation to the Intervention section:<br>"The time that the pharmacists varied by CCG, depending on the resourcing level of each of the CCG Medicines Optimisation Team. For example, we know some localities were very well resourced in terms of numbers of CCG pharmacists whereas other localities had very little CCG pharmacist resource."	lines 183-186	lines 165-168

39		2. Specification of indicators/outcome measures: Having the condition “no gastroprotection” in the denominator means that desirable corrections in prescribing lead to reductions in both the denominator (i.e. prescription of gastroprotection) and numerator (stopping of offending NSAIDs, antiplatelets, OACs) rather than just the numerator. This makes the findings difficult to interpret. It would be desirable that the authors provide additional analyses, in which the condition “no gastroprotection” is in the numerator (e.g. Denominator: patient with risk factor x; Numerator: denominator patient is prescribed offending drug without gastroprotection).	We understand that the contruction of these indicators make the interpretation more challenging. However the indicators that were used were consistent with those used for the intervention. In our further work we have removed the gastroprotection from the denomator. We are not able to recalculate the numerators and denominators in this data set as we only have the data at numerator and denominator level available for analysis.<br><br>Please see also our response to comment 54, which includes additional text we have added to the 'limitations' section of the paper.	lines 535-541	lines 459-465
40		3. The authors should make clear whether the outcome measures were pre-specified.	We have replace the word "We used" in the first line of the outcomes section with "We pre-specified"	line 206	line 188
41		4. “Not all practices contributed data at all 16 time points, as this was dependent on when the data 156 extraction was carried out. For most practices, retrospective data extraction was carried out during October 2017 and November 2017.“ The authors should provide details on how many practices contributed to which time points and reflect on whether the missing data can be assumed to be missing at random.	The number of practices included at each time point is included in table S2.<br><br>Table S2 defines the number of practices contributing to each quarter.<br>We have also added to the results section:<br>"The missing data at follow up does not seem to be related to the practice characteristics (Table 2). "	lines 313-314	lines 286-287
42		5. “Where there were quarters at the extremes of the data collection period with very few practices a pragmatic decision was taken prior to any formal analysis to exclude these from the analysis. The number of practices included in each quarter aligned by implementation quarter are presented in the supporting information (Table S1).” The authors should summarise the occurrence of missing data points in the results section and reflect on any bias this may have introduced in the discussion	We attempted to describe this in the results section, but in response to comment number 14 we have moved this to earlier in the results section, which hopefully makes this clearer.<br><br>We investigated this potential bias by rerunning the analysis with only the practices with over 6 months of follow up data, which gave very similar results. We have added the underlined text to the discussion section to try to make this clearer in the text-'However, a comparison between the rates for the raw data pre implementation for the full data set and separately for those practices contributing and not contributing data at 6 and 12 months post intervention (S6 Appendix), showed similar rates.'	line 284<br>Figure 2<br>S7 Appendix<br><br>lines 531-533	line 312<br>Figure 2<br>S7 Appendix<br><br>lines 455-458
43		6. “the assumption that practices became exposed to the intervention at the time the intervention was introduced (defined as the time when the 171 first computer search was conducted in each practice to identify patients at risk).” The authors should explain how the date of first computer search was ascertained	We have added a paragraph to the statistical methods section: "The assumption was also made that the intervention start date was the date that practices uploaded their data to CHART Online as part of the rollout of the intervention. This date was then used to indicate the implementation date for the retrospective data collection. "	lines 245-248	lines 220-223
44		7. “all pre-intervention quarters were assigned a single reference level for assessing the intervention effect. Pre-implementation base rates and numbers at risk are presented as the mean of the four quarters prior to the implementation at each site.” The analysis does therefore not seem to take into account any time trend prior to implementation, and as a result, the intervention effect may be overestimated. However, visual inspection of hazardous prescribing rates in figure 1 are somewhat reassuring for the GI bleed indicators but less so for the overall composite.	The model did explicitly include and adjust for secular trends as a standard linear trend. It is the treatment effect that is assigned to a single level - there has been no treatment and so no effect, but the outcomes do vary over that period in accordance with the secular trend.<br>We have updated the sentence to: "all pre-intervention quarters were assigned a single reference level for the treatment effect (noting that the fitted outcomes will still vary according to the fitted secular trend) "	lines 261-263	lines 236-238
45		8. The sentence “The quarter during which the intervention began, which was only partially within the intervention period, was treated as the first post-intervention quarter.” I do not understand the highlighted phrase: What is the intervention period? How can a quarter only partially fall into this period? Please, explain this better.	Updated the sentence: The quarter during which the intervention began was treated as the first post-intervention quarter. The intervention will have occurred at some point during this quarter	lines 264-267	lines 239-241
46	Results	9. LL 215 following: the numbers do not seem to add up (unless there were pilot practices which closed): Pincer was implemented in 370 practices, 21 practices either closed or were involved in piloting – therefore 350 practices were analysed. 370-21 = 349 (not 350 as stated in the manuscript)	Apologies for error - should be 349	line 300	line 273
47		10. Table 2: Care quality commission rating (the numbers of study practices should be shown)	the numer of practices added in brackets next to percentages.	line 329	line 301
48		11. Table 4: At each time point, the numerator and denominator should be presented as well as the number of practices contributing to each of those time points. This would help the reader interpret the findings better (see comment 1)	Added<br>See comment 8 and 37.	1) lines 371-374 and 377-381	1)lines 343-346 and 349-353
49		12. Supplement S4: Please, also present the rates of hazardous prescribing before and after the intervention. This is necessary to examine the relevance of any pre-intervention trends	The raw and fitted rates for pre intervention, 6 months and 12 months post intervention are presented in Table 4.	See table 4 line 385	See table 4 line 354
50		13. “At 6 months follow up, data for 293 212 practices were collected and by 12 months follow up, this had reduced to 70 (Table S2)” Please, provide data to compare baseline rates of hazardous prescribing among those included/not included in the 6 months and 12 months follow-up to explore differential loss to follow up	We have added a table (S6 Appendix)) to the supplementary materials to include the raw and adjusted pre implementation rates for those practices with up to 6 months and up to 12 months follow up.<br><br>We also added the following text to the results section:<br>"We also compared the raw and adjusted pre implementation rates for those practices with up to 6 months and up to 12 months follow up (S6 Appendix). The rates for each group of practices were found to be similar."	lines 319-321	lines 292-294

51	Discussion	14. "The greatest reductions in hazardous prescribing were apparent for the indicators where an ulcer healing 306 drug had not been prescribed when there was a medication-related increased risk of gastrointestinal 307 bleeding (Indicators A, B, C, E and F)." The way that the indicators were specified, it would be more correct to say: The greatest reductions were apparent for indicators targeting hazardous prescribing of NSAIDs or antiplatelets in patients with risk factors for gastrointestinal bleeding who were not also prescribed an ulcer healing 306 drug (Indicators A, B, C, E and F).	Thank you. This is a really helpful suggestion and we have changed in the text as recommended.<br><br>Whilst reviewing we also noticed 2 indicators which aslo had a significant reduction in hazardous prescribing, so we have made an additional amendment. Text amended "The only other indicators where a statistically significant reduction in hazardous prescribing was observed to be associated with the intervention was for the prescription of a non-steroidal anti-inflammatory drug (NSAID) to a patient with heart failure (Indicator I) at 6 months (but not 12 months) post-intervention, and for the prescription of a NSAID to a patient with chronic kidney disease (Indicator K) at 12 months, post-intervention."	lines 431-434<br><br>lines 438-443	lines 379-381<br><br>lines 381-386
52		15. "We did, however, take account of secular trends and this was clearly important given the trend towards safer 324 prescribing over time (see Figure 1)." The authors should explain in more detail here, whether, how and why their accounting for pre-intervention trends differed from conventional approaches of segmented regression of time series and how this may have influenced their findings	Added to the strengths and limmitations section "The model used was an extension of the conventional segmented regression which allows explicitly for the intervention effect to be non-linear. We expected a priori when we specified the analysis that the treatment effect would reduce over time and wanted a model that would capture the time trends here. We were fortunate in having a very large sample size to allow a rich representation (via a simple qualitative post-intervention time parameter) of these trends."	lines 519-523	lines 447-451
53		16. "However, a sensitivity analysis showed similar findings when analysing practices with at least six months of follow-up data" As the authors state in the results section, it is to be expected that restricting analysis to practices who drove the findings of the primary analysis insufficiently addresses the concern that follow up data was available from only 212/350 (61%) practices at 6 months and 70/350 (20%) of practices at 12 months. More efforts should be made to explore, whether loss to follow up was differential, e.g. by comparison of pre-intervention data (for the 6 months follow up data) and 6 months data (for the 12 months follow up) for practices with and without follow up data at each data point.	Added the text to the results section:<br>"The missing data at follow up does not seem to be related to the practice characteristics (Table 2). We looked at the characteristics of the 212 practices included at 6-month post intervention and the 70 practices included at 12 months post intervention and found these to be comparable with the 343 participating practices. As a sensitivity analysis, we also repeated the analysis including only practices with at least 6 months of follow up data. A similar effect size was noted at both 6- and 12-months post-intervention (S5 Appendix). This was expected as the intervention effect estimates using the entire data set were derived from those practices that had sufficient follow-up. We also compared the raw and adjusted pre implementation rates for those practices with up to 6 months and up to 12 months follow up (S6 Appendix). The rates for each group of practices were found to be similar."	lines 313-321	lines 286-294
54		17. row 366 "Overall, these studies confirm that these complex but pragmatic interventions can be effective at reducing 366 hazardous prescribing, but the effects are greatest where a straightforward action can be taken (such as prescribing an ulcer-healing drug to a patient at risk of medication-related gastro-intestinal bleed) compared with stopping (or changing) a medication that may be perceived to be providing benefit to a patient, despite the risk (such as prescribing a beta-blocker in a patient with asthma)." It is not clear from the data provided, whether changes in hazardous prescribing were due to stopping of NSAIDs or prescription of gstroprotection. The way that the indicators were specified, the prescription of gastroprotection would lead to reductions in the denominator and it is unclear what this would mean for the numerator. From the data, it therefore remains unclear to which extent reductions were driven by initiation of gastroprotection, stopping of NSAIDs/antiplatelets or both.	As noted above, in relation to comment 39, we acknowledge that the way that the indicators have been constructed means that taking an action such as adding a proton pump inhibitor removes a patient from the numerator and denominator for subsequent data collections. Nevertheless, as the denominator is always larger than the numerator, this still means that reductions in both leads to reduction in the proportion of patients exposed to hazardous prescribing. We have only numerator and denominator data and so we are not able to tell whether a drug has been stopped (NSAID) or a PPI added in realtion to indicators A and B, although in relation to indicators C, E and F it is most likely that a PPI would have been added rather than stopping medications used in secondary prevention.<br><br>We have added the following text to the limitations part of the discussion: "We constructed our indicators so that the denominators contained 'at-risk' groups of patients but acknowledge that in some cases taking an action such as adding a proton pump inhibitor removes a patient from both the numerator and denominator for subsequent data collections. Given that the denominator is always larger than the numerator, this still means that reductions in both leads to reduction in the proportion of patients exposed to hazardous prescribing. Nevertheless, a larger effect might have been demonstrated if had put the term 'without co-prescription of an ulcer healing drug' in the denominator".	lines 535-541	lines 459-465
55	Conclusion	18. "The greatest reductions in hazardous 415 prescribing were for indicators associated with risk of gastrointestinal bleeding, particularly where prescription of an ulcer-healing drug would improve patient safety." Please, see comments 2, 14 and 17 [this refers to comments from reviewer TD]	Even though we cannot be certain of the changes made to reduce hazardous prescribing (i.e. stopping a drug vs prescribing an ulcer-healing drug) the indicators that showed greatest reductions were those where prescribing an ulcer-healing drug was an option (A, B, C, E, F), but there was no significant effect for indicator D where the anticpated action would have been to stop an NSAID. Therefore we think this conclusion holds and have not changed the wording.		
56	Comments from reviewer 5:	Reviewer #5: The study uses an interrupted time series approach to evaluate the rate of hazardous prescribing after implementing the PINCER intervention. I found this to be an interesting and generally well presented study. My comments mainly concern the detail given on the statistical model used and its assumptions.	See below	n/a	n/a
57		- Although the statistical model is described in words, I think it would be beneficial to also provide the model explicitly, perhaps in the supplementary material. This is not a straightforward model and would be clearer written out in equation form with full details given.	The model has been added to the supporting information	S2	S2

58	- Is it assumed that there is no time-dependant trend prior to intervention? The definition of base rates as an average of four quarters prior to implementation suggests it may be, although, again, this would be clearer if the model was written out. Is this a reasonable assumption? Could the data be plotted where the x-axis is 'quarter relative to intervention' (possibly after accounting for secular trend)?	The model does explicitly account for secular trends - sorry if this was not clear; we have added more explanation in the revised draft to make this explicit.<br>We have added the explicit model to the supporting information, so hopefully this will help to make it clearer.<br>We have also added the sentence: "all pre-intervention quarters were assigned a single reference level for the treatment effect (noting that the fitted outcomes will still vary according to the fitted secular trend)" to the Statistical Methods section.<br>The OR are presented relative to the intervention quarter in charts in Figure 2 and the supporting information S5.	lines 261-263	lines 236-238
59	- Another assumption of the interrupted time series model is that there is no residual autocorrelation. Is this justifiable? Could this be commented on and/or tested?	We have added the following text to the Strengths and limitations section" In the interrupted time series model, we have assumed that there is no residual autocorrelation. This assumption is implicit and unfortunately hard to test within the modelling framework/software we have used. A priori we expected that autocorrelation within practices would not be a substantive issue as the events of concern are intrinsically independent. We did anticipate strong correlations within practices and accounted for these in the model specification and also by utilising robust SE estimates."	lines 549-553	lines 471-475
60	- Is it possible that a patient was at risk of or exposed to more than one hazardous prescribing indicator? The descriptions in Table 1 and the data in Table 3 suggests that this may be the case. Has any overlap been accounted for when calculating the numerators and denominators of the composite indices?	Patients my occur in multiple indicators, but in the analysis, the indicators we treated as separate 'errors', so the composite indicators use the total errors, not patients.	n/a	n/a
Additional amendments	1. Please ensure that the Author List in your manuscript file matches the Author List in the online submission form exactly. Authors should be listed in the same order, and the order of individual first and last author names must be identical in both locations.	Thank you- Updated	lines 5-7	lines 5-7
	2. Please amend your detailed Financial Disclosure statement. This is published with the article. It must therefore be completed in full sentences and contain the exact wording you wish to be published.<br>- Please clarify all sources of funding (financial or material support) for your study. List the grants (with grant number) or organizations (with url) that supported your study, including funding received from your institution.<br>- State the initials, alongside each funding source, of each author to receive each grant.<br>- State what role the funders took in the study. If the funders had no role in your study, please state: "The funders had no role in study design, data collection and analysis, decision to publish, or preparation of the manuscript."<br>- Please ensure that the funders and grant numbers match between the Financial Disclosure field and the Funding Information tab in your submission form. Note that the funders must be provided in the same order in both places as well.	Original text:<br>Health Foundation, London England; East Midlands Academic Health Science Network, Nottingham, England. DMA was supported by the National Institute for Health Research (NIHR) Greater Manchester Patient Safety Translational Research Centre (award number: PSTRC-2016-003). The views expressed are those of the authors and not necessarily those of the Health Foundation, East Midlands AHSN, NIHR or the Department of Health and Social Care. KK is supported by the National Institute for Health Research (NIHR) Applied Research Collaboration East Midlands (ARC EM) and the NIHR Leicester Biomedical Research Centre (BRC).<br><br>Changed to:<br>This study was funded by The Health Foundation (www.health.org.uk, Award Number: 7419) AJA, DMA, MJB, RAE, KK, SR, AS and ANS, and the East Midlands Academic Health Science Network (www.emahsn.org.uk, Award Number: 39701) AJA, SR.<br>DMA was supported by the National Institute for Health Research (NIHR) Greater Manchester Patient Safety Translational Research Centre (award number: PSTRC-2016-003, http://www.patientsafety.manchester.ac.uk).<br>KK is supported by the National Institute for Health Research (NIHR) Applied Research Collaboration East Midlands (https://arc-em.nihr.ac.uk) and the NIHR Leicester Biomedical Research Centre (https://www.leicesterbrc.nihr.ac.uk).<br>The funders had no role in study design, data collection and analysis, decision to publish, or preparation of the manuscript.	Financial Disclosure statement and Funding Information tab in your submission form	Financial Disclosure statement and Funding Information tab in your submission form
	3. We have amended your Competing Interest statement to include a competing interest declared by your co-author, Dr. Aloysius Niroshan Siriwardena. Please review the added statement below and let us know if anything is incorrect.<br>"Funding from National Institute for Health Research for several unrelated studies listed in publication."	Thank you for updating this text- all correct.	n/a	n/a
	4. We do not publish any copyright or trademark symbols that usually accompany proprietary names, eg (R), (C), or TM (e.g. next to drug or reagent names). Therefore please remove all instances of trademark/copyright symbols throughout the text, including ® in the manuscript.	Removed from Table 2	Table 2	Table 2
