# Supplementary material for: Scaling-up a pharmacist-led information technology intervention (PINCER) to reduce hazardous prescribing in general practices: Multiple interrupted time series study
Source: PLoS Med. 2022 Nov 16;19(11):e1004133. doi: 10.1371/journal.pmed.1004133 (PMC9718399; doi:10.1371/journal.pmed.1004133)
Supplement: Attachment — Submitted filename: Response to reviewers Oct 2022.pdf [file pmed.1004133.s010.pdf]

comment reference number	reviewer/ section	Reviewers comments	Authors response	location of amendment - tracked version	location of amendment - clean version
1	Abstract:	a) In the last sentence of the Abstract "Methods and Findings section," please describe the main limitation(s) of the study's methodology. You indicate that this has been added; please ensure that you have situated in the right place within the abstract. It looks like you added the statement to the Abstract's Conclusions – please move it to the "Methods and findings" instead.	Moved as requested from 'Conclusions' to 'Methods and Findings'.	row 58-61	58-61
2	Abstract:	b) Please interpret the study based on the results presented in the abstract, emphasizing what is new without overstating your conclusions. In addition please state specific implications of your study	We have updated the conclusion and have included a specific implication: "The PINCER intervention, when rolled out at scale in routine clinical practice, was associated with a reduction in hazardous prescribing by 17% and 15% at 6- and 12-months post-intervention. The greatest reductions in hazardous prescribing were for indicators associated with risk of gastrointestinal bleeding. These findings support the wider national rollout of PINCER in England."	row 65-68	64-67
3	Author summary:	a) Please put the limitations statement under the subheading "What Did the Researchers Do and Find?"	Moved limitations bullet point from 'what do these findings mean?' to 'What Did the Researchers Do and Find?'	row 97, 98	91-92
4	Author summary:	b) Author summary - Please avoid vague statements such as "these results have major implications for policy/clinical care". Mention only specific implications substantiated by the results.	The following specific implication has been added to the conclusion: "These findings support the wider national rollout of PINCER in England." We have also separated the first bullet point into two points for clarity.	row 101- 103	94-97
5	Strobe checklist	Thank you for providing a completed STROBE checklist. Please remove the Column, Line No, as this will likely change in the event of publication. Instead provide the paragraph number in addition to the section number.	Completed with Paragraph numbers.		
6	Disussion	In response to comment 54, the authors now state that: "Nevertheless, a larger effect might have been demonstrated if had put the term "without co-prescription of an ulcer healing drug in the denominator". The word denominator should be replaced by the word numerator.	Thank you, updated in comment 54 and in the discussion section of the paper. We also noticed the missing word 'we' in this sentence, so this has also been added.	row 475-476	467-468

Additional amendment	Abstract	Additional amendment	On reviewing the abstract, we believe that it would be useful to specify that the practices were all in the East Midlands. Therefore, we made an additional edit to add the following text to the 'Methods and Findings' section of the appendix. We added the text: "in the East Midlands region of England".	row 45	row 45
	Author list	Additional amendment	We have decided to make Sarah Rodgers and Amelia Taylor joint first authors. With guidance from PlosMedicne staff, we have made the following amendments:<br>Added the & to the author list 'Sarah Rodgers,1,2 & Amelia C Taylor*,1 .'<br>Added ' & joint first author' after the Corresponding Author's email.	row 5 and 21	row 5 and 21
