# Supplementary material for: Scaling-up a pharmacist-led information technology intervention (PINCER) to reduce hazardous prescribing in general practices: Multiple interrupted time series study
Source: PLoS Med. 2022 Nov 16;19(11):e1004133. doi: 10.1371/journal.pmed.1004133 (PMC9718399; doi:10.1371/journal.pmed.1004133)
Supplement: Attachment — Submitted filename: Review TD.docx [file pmed.1004133.s008.docx]

**Overall assessment**

The authors are to be congratulated for conducting and reporting efforts to scale up an intervention, which has been shown to be effective under the conditions of a randomised trial. Their findings indicate improvements in their chosen endpoints, but there are three main concerns that the authors should address prior to publication:

- The way that the indicators on GI risk are specified, desirable changes in prescribing (i.e. initiation of gastroprotection) would lead to reductions in the denominator, which makes changes difficult to interpret. As a minimum, the authors should provide numerator and denominator data for all data points
- There has been substantial loss to follow up and the potential for bias resulting from this should be explored further by comparison of baseline data for those with and without loss to follow up
- The analysis methods appear pragmatic but their accounting for pre-intervention trends seems to fall short of conventional segmented regression of time series data. As a minimum, the authors should provide preintervention trends of rates of hazardous prescribing for all indicators

**Methods**

1. *“Third, the pharmacists (sometimes supported by pharmacy technicians) work with, and support, general 131 practice staff to implement the agreed action plan.”* There is insufficient detail to understand what the pharmacist contribution comprised. Please, provide more detail as to the pharmacist time dedicated to PINCER work as well as the nature of their “support” to practice staff, e.g.

- *Were the pharmacists present in the practices over the whole implementation period?*
- *If not, how long were they present? If so, what was the full time equivalent they dedicated to PINCER work?*
- *Did the pharmacists provide input into individual patient care?*
- *Did they access individual patient records?*
- *Did pharmacists see patients?*
- *Did pharmacists only advise on or help amend processes at practice level?*

2. Specification of indicators/outcome measures: Having the condition “no gastroprotection” in the denominator means that desirable corrections in prescribing lead to reductions in both the denominator (i.e. prescription of gastroprotection) and numerator (stopping of offending NSAIDs, antiplatelets, OACs) rather than just the numerator. This makes the findings difficult to interpret. It would be desirable that the authors provide additional analyses, in which the condition “no gastroprotection” is in the numerator (e.g. Denominator: patient with risk factor x; Numerator: denominator patient is prescribed offending drug without gastroprotection).
3. The authors should make clear whether the outcome measures were pre-specified.
4. “Not all practices contributed data at all 16 time points, as this was dependent on when the data 156 extraction was carried out. For most practices, retrospective data extraction was carried out during October 2017 and November 2017.“ The authors should provide details on how many practices contributed to which time points and reflect on whether the missing data can be assumed to be missing at random.
5. “Where there were quarters at the extremes of the data collection period with very few practices a 174 pragmatic decision was taken prior to any formal analysis to exclude these from the analysis. The number 175 of practices included in each quarter aligned by implementation quarter are presented in the supporting 176 information (Table S1).” The authors should summarise the occurrence of missing data points in the results section and reflect on any bias this may have introduced in the discussion
6. “the assumption that practices became exposed to the intervention at the time the intervention was introduced (defined as the time when the 171 first computer search was conducted in each practice to identify patients at risk).” The authors should explain how the date of first computer search was ascertained
7. *“all pre-intervention 185 quarters were assigned a single reference level for assessing the intervention effect. Pre-implementation 186 base rates and numbers at risk are presented as the mean of the four quarters prior to the 187 implementation at each site.*” The analysis does therefore not seem to take into account any time trend prior to implementation, and as a result, the intervention effect may be overestimated. However, visual inspection of hazardous prescribing rates in figure 1 are somewhat reassuring for the GI bleed indicators but less so for the overall composite.
8. The sentence “The quarter during which the intervention began, which was only partially 188 within the intervention period, was treated as the first post-intervention quarter.” I do not understand the highlighted phrase: What is the intervention period? How can a quarter only partially fall into this period? Please, explain this better.

**Results**

9. LL 215 following: the numbers do not seem to add up (unless there were pilot practices which closed): Pincer was implemented in 370 practices, 21 practices either closed or were involved in piloting – therefore 350 practices were analysed. 370-21 = 349 (not 350 as stated in the manuscript)
10. Table 2: Care quality commission rating (the numbers of study practices should be shown)
11. Table 4: At each time point, the numerator and denominator should be presented as well as the number of practices contributing to each of those time points. This would help the reader interpret the findings better (see comment 1)
12. Supplement S4: Please, also present the rates of hazardous prescribing before and after the intervention. This is necessary to examine the relevance of any pre-intervention trends
13. “At 6 months follow up, data for 293 212 practices were collected and by 12 months follow up, this had reduced to 70 (Table S2)” Please, provide data to compare baseline rates of hazardous prescribing among those included/not included in the 6 months and 12 months follow-up to explore differential loss to follow up

**Discussion**

14. *“The greatest reductions in hazardous prescribing were apparent for the indicators where an ulcer healing 306 drug had not been prescribed when there was a medication-related increased risk of gastrointestinal 307 bleeding (Indicators A, B, C, E and F).”* The way that the indicators were specified, it would be more correct to say: The greatest reductions were apparent for indicators targeting hazardous prescribing of NSAIDs or antiplatelets in patients with risk factors for gastrointestinal bleeding who were not also prescribed an ulcer healing 306 drug (Indicators A, B, C, E and F).
15. *“We did, 323 however, take account of secular trends and this was clearly important given the trend towards safer 324 prescribing over time (see Figure 1).”* The authors should explain in more detail here, whether, how and why their accounting for pre-intervention trends differed from conventional approaches of segmented regression of time series and how this may have influenced their findings
16. *“However, a sensitivity 329 analysis showed similar findings when analysing practices with at least six months of follow-up data”* As the authors state in the results section, it is to be expected that restricting analysis to practices who drove the findings of the primary analysis insufficiently addresses the concern that follow up data was available from only 212/350 (61%) practices at 6 months and 70/350 (20%) of practices at 12 months. More efforts should be made to explore, whether loss to follow up was differential, e.g. by comparison of pre-intervention data (for the 6 months follow up data) and 6 months data (for the 12 months follow up) for practices with and without follow up data at each data point.
17. *“Overall, these studies confirm that these complex but pragmatic interventions can be effective at reducing 366 hazardous prescribing, but the effects are greatest where a straightforward action can be taken (such as 367 prescribing an ulcer-healing drug to a patient at risk of medication-related gastro-intestinal bleed) 368 compared with stopping (or changing) a medication that may be perceived to be providing benefit to a 369 patient, despite the risk (such as prescribing a beta-blocker in a patient with asthma).”* It is not clear from the data provided, whether changes in hazardous prescribing were due to stopping of NSAIDs or prescription of gstroprotection. The way that the indicators were specified, the prescription of gastroprotection would lead to reductions in the denominator and it is unclear what this would mean for the numerator. From the data, it therefore remains unclear to which extent reductions were driven by initiation of gastroprotection, stopping of NSAIDs/antiplatelets or both.

**Conclusion**

18. *“The greatest reductions in hazardous 415 prescribing were for indicators associated with risk of gastrointestinal bleeding, particularly where 416 prescription of an ulcer-healing drug would improve patient safety.” Please, see comments 2, 14 and 17*
